# A Brief Introduction to Chemical Reaction Optimization

**DOI:** 10.1021/acs.chemrev.2c00798

**Published:** 2023-02-23

**Authors:** Connor J. Taylor, Alexander Pomberger, Kobi C. Felton, Rachel Grainger, Magda Barecka, Thomas W. Chamberlain, Richard A. Bourne, Christopher N. Johnson, Alexei A. Lapkin

**Affiliations:** † Astex Pharmaceuticals, 436 Cambridge Science Park, Milton Road, Cambridge CB4 0QA, U.K.; ‡ Innovation Centre in Digital Molecular Technologies, Department of Chemistry, University of Cambridge, Lensfield Road, Cambridge CB2 1EW, U.K.; § Department of Chemical Engineering and Biotechnology, University of Cambridge, Philippa Fawcett Drive, Cambridge CB3 0AS, U.K.; ∥ Chemical Engineering Department, Northeastern University, 360 Huntington Avenue, Boston, Massachusetts 02115, United States; ⊥ Chemistry and Chemical Biology Department, Northeastern University, 360 Huntington Avenue, Boston, Massachusetts 02115, United States; # Cambridge Centre for Advanced Research and Education in Singapore, 1 Create Way, 138602 Singapore; ∇ Institute of Process Research and Development, School of Chemistry and School of Chemical and Process Engineering, University of Leeds, Leeds LS2 9JT, U.K.

## Abstract

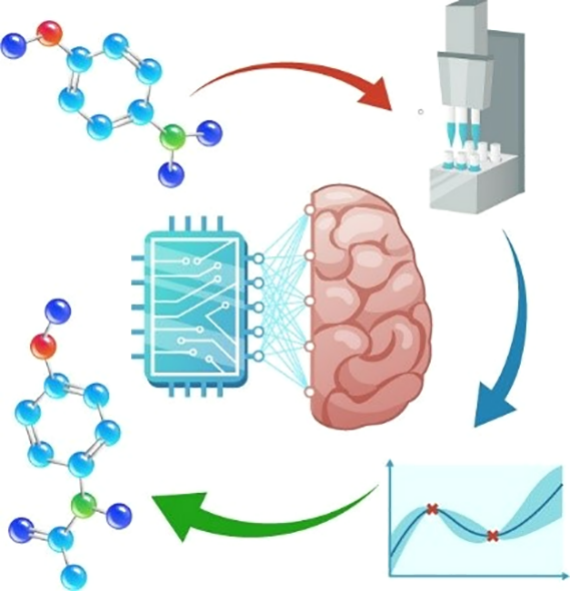

From the start of a synthetic chemist’s training, experiments are conducted based on recipes from textbooks and manuscripts that achieve clean reaction outcomes, allowing the scientist to develop practical skills and some chemical intuition. This procedure is often kept long into a researcher’s career, as new recipes are developed based on similar reaction protocols, and intuition-guided deviations are conducted through learning from failed experiments. However, when attempting to understand chemical systems of interest, it has been shown that model-based, algorithm-based, and miniaturized high-throughput techniques outperform human chemical intuition and achieve reaction optimization in a much more time- and material-efficient manner; this is covered in detail in this paper. As many synthetic chemists are not exposed to these techniques in undergraduate teaching, this leads to a disproportionate number of scientists that wish to optimize their reactions but are unable to use these methodologies or are simply unaware of their existence. This review highlights the basics, and the cutting-edge, of modern chemical reaction optimization as well as its relation to process scale-up and can thereby serve as a reference for inspired scientists for each of these techniques, detailing several of their respective applications.

## 1 Introduction

Chemical reaction optimization is a term that has a variety of meanings depending on the chemist defining it, with a large corresponding variance in expectations of optimization capability and proficiency. As reaction optimization is largely unexplored during undergraduate chemistry teaching,^1−3^ many research chemists are simply unaware of existing optimization techniques and are therefore unlikely to employ robust strategies in their workflows as their career progresses. This is particularly true in academic research, where intuition-based optimization^4−12,^ is commonplace despite the increasing evidence showing that reaction modeling and algorithmic optimizations are more efficient in relation to both time and material and therefore cost.^13−16^ For these reasons, the use of these methodologies is much more widespread in industrial research and development, particularly in process laboratories compared to their discovery laboratory counterparts,^17,18^ as manufacturing conditions often result from vigorous optimization protocols. Consequently, there is often a large disparity in the familiarity of optimization techniques between industrial and academic researchers, particularly because industrial scientists also often have internal multidisciplinary teams of statisticians and process chemists to collaborate with. However, the techniques covered are not inaccessible for chemists, and increasing the exposure of these methodologies will make them more widespread across both academic research and teaching, thereby enriching the skillset of the entire chemical community. Although the primary aims of many scientists (particularly synthetic chemists) may not be to achieve truly optimal processes, familiarity with the concepts discussed herein will help researchers meet the needs of the modern and evolving laboratory.

This review aims to critically analyze and compare major chemical reaction optimization techniques, thereby helping to deliver an accessible account of optimization strategies (with references to their applications) for the general chemical scientist. As many of these methodologies borrow concepts from related fields, such as statistics, computer science, process chemistry, and engineering, this review will help to diversify the chemist’s toolkit and serve as a comprehensible reference for optimization campaigns. Although reaction optimization is often related to reaction yields, it may also be performed with respect to purity, *E*-factor, enantiomeric excess, etc., and these concepts will be explored further. Typical reaction variables that are optimized are also often described as either continuous (in a numeric form, such as temperature or reaction time) or categorical (discrete options, such as solvent or catalyst/ligand choice). Further in-depth reading will also be provided at each stage for interested scientists seeking a deeper understanding of the workings of each methodology. We also discuss how to explore the generated reaction knowledge within the subsequent process scale-up efforts. By providing tools for considerations of scale-up challenges and complexity in the early stages of process optimization, we hope to help chemists to guide their optimization efforts toward scalable processes and thus facilitate the translation of critical laboratory discoveries into commercially available products.

## 2 One Factor At a Time (OFAT)

“Intuition-based optimization” largely relates to optimization using the trends and anecdotal observations from experienced chemists to improve reaction metrics. Alongside optimization via chemical intuition, one factor at a time (OFAT) approaches often substitute as a method for chemical process optimization and understanding.^16,19^ This is primarily performed in academia and in the presence of a structured, yet simple to follow, procedure, making this technique seem both effective and accessible. The OFAT methodology itself requires some scientific intuition, where experiments are iteratively performed by fixing all process factors except for one. After the best value for the one factor has been identified, that value is fixed while another set of experiments are executed to optimize another factor until each factor is optimized and the scientist believes that they have arrived at the optimum reaction conditions.^20^ These factors can be any number of experimental conditions (such as temperature, stoichiometry, reaction time, etc.) which, when combined, constitute a multidimensional space with many possible combinations of factors to make up one experiment. This is termed the parameter space and is constrained by the upper and lower limits of each factor (for example, max and min temperature).

**Figure 1:**
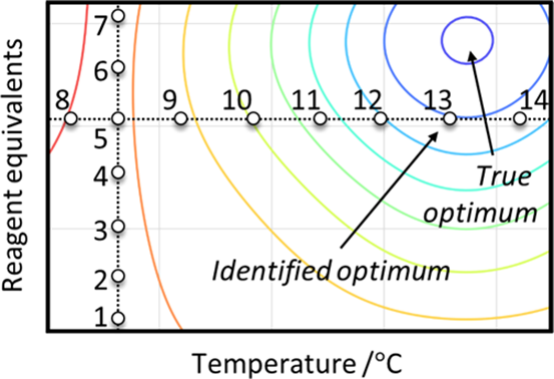
An example of an OFAT experimental procedure in varying temperature and reagent equivalents, where ○ represents a numbered experimental data point and the blue region indicates the true optimum area of parameter space. Response surface is contoured from red (low response) to blue (high response).

The OFAT approach is often inaccurate and inefficient as an optimization technique, and the method frequently misinterprets the chemical process as there are no considerations for any synergistic effects between the factors considered.^21^ Interactions between the experimental factors are ignored, as this linear experimental procedure is applied to chemical reaction outputs that give exclusively nonlinear responses.^22^ This nonlinearity can be explained by statistical or physical modeling but is not explored using OFAT, which therefore often incorrectly identifies true optimal reaction conditions.^23,24^ An exemplar schematic of an OFAT campaign is shown in Figure 1, mapped onto the response surface for a given parameter space for a chemical reaction (in which reagent equivalents and temperature are considered). The optimization is initialized as the temperature is fixed, and iterative experiments (1–7) are performed to identify the optimum reagent equivalents. After this value is found at experiment 5, subsequent experiments are performed (8–14) to determine the optimum temperature. As there are only two factors considered in this example, the optimization is now complete with a set of experimental conditions found that are presumed to be optimal. However, as the response surface cannot be known a priori for a real chemical example, it is difficult to estimate the distance from this identified set of optimum conditions and the true optimum for the system.

**Scheme 1:**
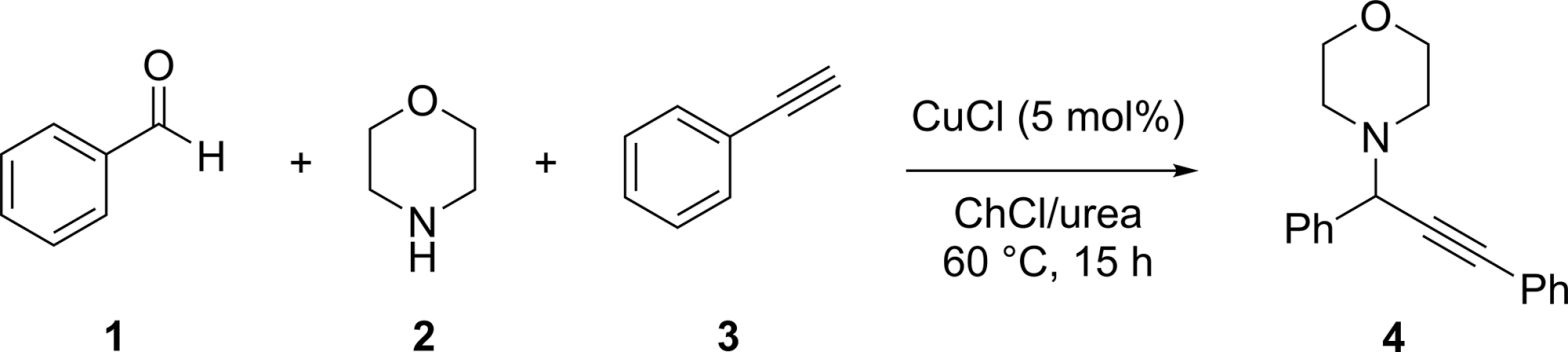
Model Reaction Used for OFAT Optimization for the Synthesis of Propargylamine Derivatives^25^

Generally, scientists perform OFAT campaigns as the method can be performed without mathematical modeling, which is a critical advantage when performing lab-based experiments and is one major reason it is prevalent. A recent example by Abtahi and Tavakol^25^ shows the use of OFAT optimization to achieve fair yields in the synthesis of bioactive propargylamine scaffolds. The model reaction was optimized, shown in Scheme 1, and the identified reaction conditions were then applied to several substrates achieving 38–91% yield. The optimization procedure began by fixing the temperature and reaction time and optimizing the reaction media and catalyst to obtain the highest reaction yield. The reaction media and catalyst were then fixed as the temperature and reaction time were optimized, followed by a fixing of all factors except catalyst loading, as this factor was finally optimized to achieve a 75% yield in the model reaction.

There are several examples from the literature of this technique’s application to various chemistries, in many cases with different levels of applied chemical intuition but still following the structure of the OFAT methodology.^26−28^ In our own laboratories, when undergraduate chemists are given the task of optimizing a reaction, students will often employ OFAT techniques as they are unaware of other means of optimization.^29^ This is not the fault of the student, nor is it the fault of the academics optimizing their reactions in this way, as this does perform as a rudimentary technique to achieve improved reaction yields. However, as research laboratories are beginning to diversify their equipment by incorporating advanced technologies such as automated retrosynthesis software and experimentation,^30,31^ it is also important for chemists to evolve at the same pace by diversifying their own skillsets to fully harness the capabilities of the evolving laboratory. Synthetic chemists, in recent years, have begun to embrace facets of process chemistry, chemical engineering, analytical chemistry, and computer science, to name a few.^17,18^ Concurrently, it is important to facilitate better understanding and adoption of reaction optimization methods as OFAT optimizations are superseded by more robust and more efficient techniques.^32−34^ Chemical reaction optimization by OFAT has therefore been included in this paper for comparative purposes.

## 3 Design of Experiments (DoE)

One robust and widely used optimization technique, particularly in the pharmaceutical and fine chemical industries, is design of experiments (DoE). DoE is a class of statistical methods that aim to build a model that can mathematically describe the output of a chemical reaction (such as reaction yield, purity etc.) based on the experimental inputs for that reaction (factors such as temperature or reaction time). There are many reports of widespread DoE usage for reaction optimization, but it is also often used in the literature as a comparison with OFAT optimizations to highlight its efficiency and thereby refute OFAT.^16,23^ There are three main objectives for DoE: screening, optimization, and robustness.^35^ Screening involves the identification of factors that have a significant effect on reaction output, as well as their respective upper and lower bounds. Optimization focuses on the determination of the optimum factor levels, such as the optimum temperature and reagent equivalents, to achieve the best reaction output possible. Finally, robustness testing involves the identification of the sensitivity of this response to small changes in the experimental factors; this is important on a process scale to understand how possible deficiencies in reactors may lead to suboptimal outputs.

The practical manner of running DoE campaigns focuses on performing predefined experiments from a structured experimental design. These designs are templates from which to execute experiments, based on the factors and bounds of interest, that explore the parameter space efficiently and provide data in a structured format to build robust statistical models.^20,36^ The format of this experimental data is important and often difficult to replicate/analyze using human intuition, which is why DoE software is often implemented, such as MODDE,^37^ JMP,^38^ Design-Expert,^39^ or toolbox applications in languages such as R, MATLAB, and Python. After data collection and the fitting of the statistical model, optimized process parameters can then be identified, and response surfaces are often plotted to help visualize the effects of experimental factors on the chemical output.

**Scheme 2:**
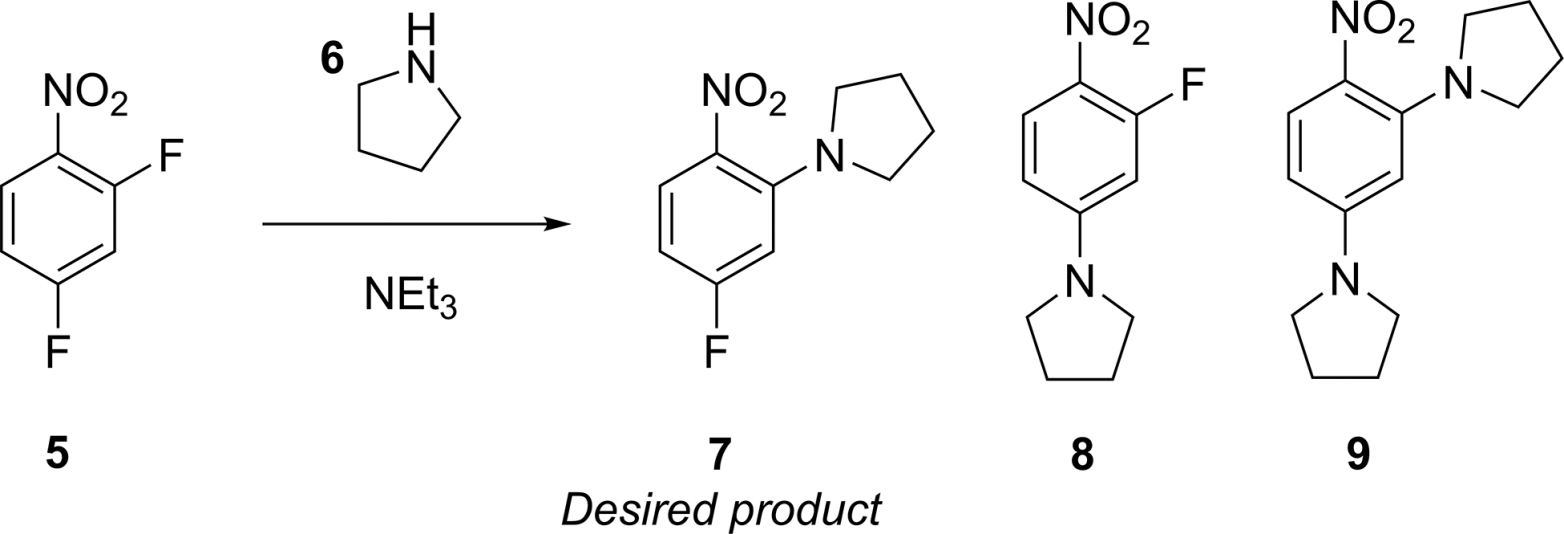
S_N_Ar System of Interest, Where the DoE Campaign Aims to Optimize the Yield of the Ortho-Substituted Product, **7**(1)

**Figure 2:**
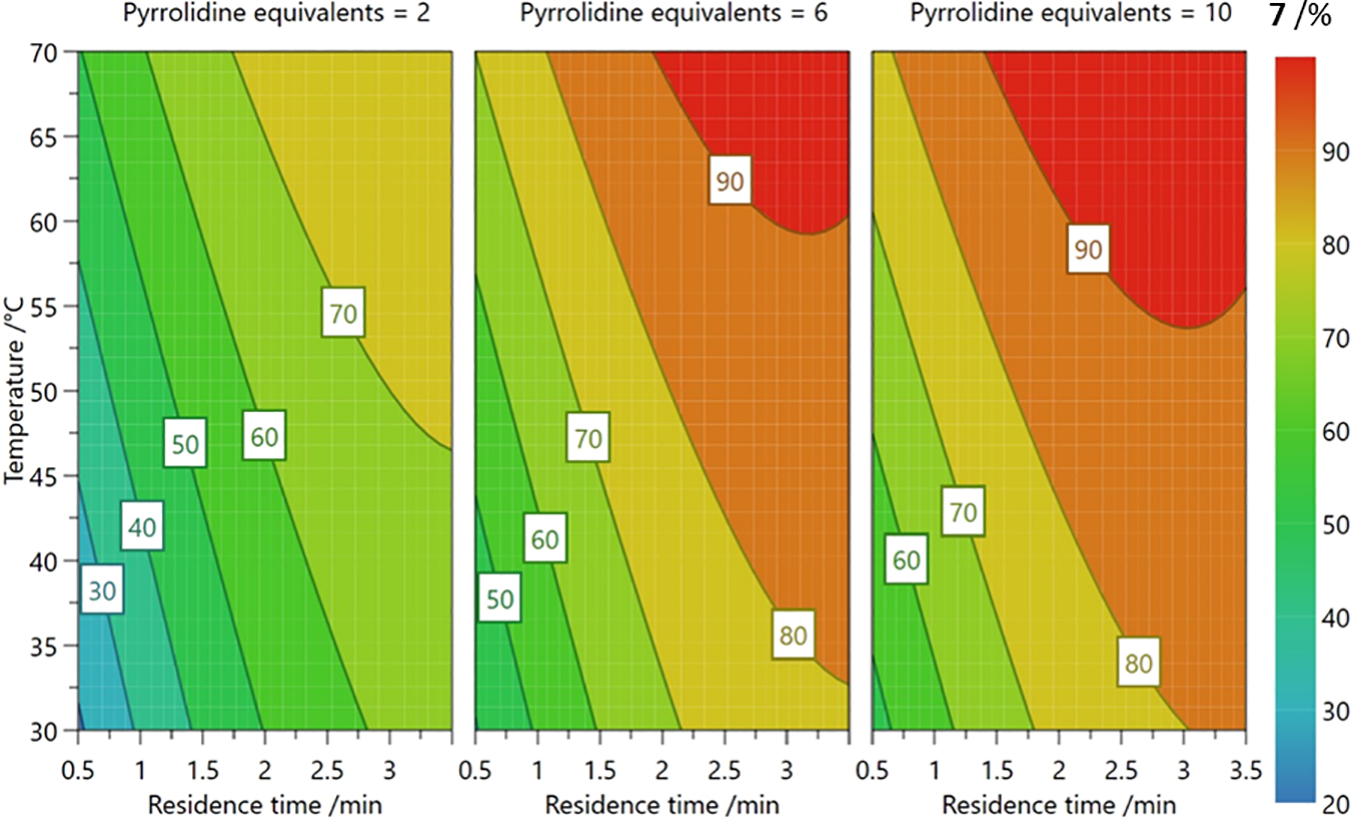
Contour plot for the response of **7**, showing how the yield of the desired product varies with respect to changing experimental conditions.^1^

One optimization campaign performed using DoE in our laboratory is the multistep S_N_Ar reaction of 2,4-difluoronitrobenzene with pyrrolidine, as shown in Scheme 2.^1^ This reaction has multiple products, but the study aimed to produce the ortho-substituted product, **7**, in the highest yield by using a face-centered central composite (CCF) design; more details on specific DoE designs are outlined in section 3.2. The 17 experiments were predefined and executed according to this design, where the experimental bounds for each of the defined factors were: residence time (0.5–3.5 min, as this was a flow experiment), temperature (30–70 °C), and equivalents of pyrrolidine (2–10). Among these experiments were three repeated center-point experiments, or replicates, that ensure that any extraneous variables are identified (uncontrolled variables that could be changing unknowingly, e.g., stock solution degradation throughout the experimental procedure). These replicates are experiments with the center value for each factor, e.g., a reaction temperature of 50 °C in this example, and are conducted throughout the course of the 17 experiment campaign. The outputs from each experiment were then inputted into the DoE software, MODDE, to identify the optimum reaction conditions that afforded the highest yield of **7**. After statistical analysis, a response surface was plotted for the chemical process to visualize the effect of each factor on the yield of our ortho-substituted product, as shown in Figure 2. It was found that the highest yield of **7** could be obtained by using higher temperature, longer residence times, and higher pyrrolidine equivalents, leading to a yield of 93%. However, these reaction conditions also produced the highest yield of the impurity, the disubstituted product, **9**. A process chemist could then use this information to decide for a particular reactor system if product throughput is more important than other downstream processes, such as product purity and separation.

**Scheme 3:**
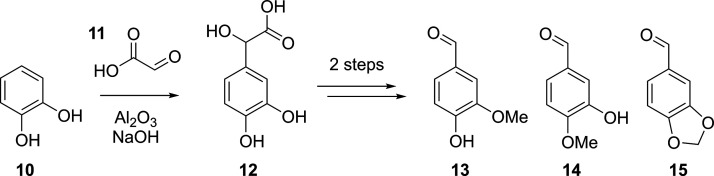
One Reaction of Interest, Optimizing the Yield and Selectivity of the Desired 3,4-Dihydroxymandelic Acid Intermediate, **12** This intermediate can then be used to synthesize either vanillin, **13**, *iso*-vanillin, **14**, or heliotropin, **15**.^40^

Another campaign highlighting the effectiveness of DoE was reported by Minisci and co-workers^40^ on the synthesis of vanillin, *iso*-vanillin, and heliotropin. Several DoE studies were employed to identify optimum process factors for each synthetic step, one of which was the initial addition of glyoxylic acid, **11**, to catechol, **10**, to form the desired 3,4-dihydroxymandelic acid intermediate, **12**, as shown in Scheme 3. Their initial attempts to reproduce reported literature led to poor selectivity and hence poor yields (<20%), so the authors systematically employed full factorial DoE designs to identify the important experimental factors and to estimate the main factor effects and interactions, hence giving an accurate statistical model for the process and thereby optimizing the product output.

**Figure 3:**
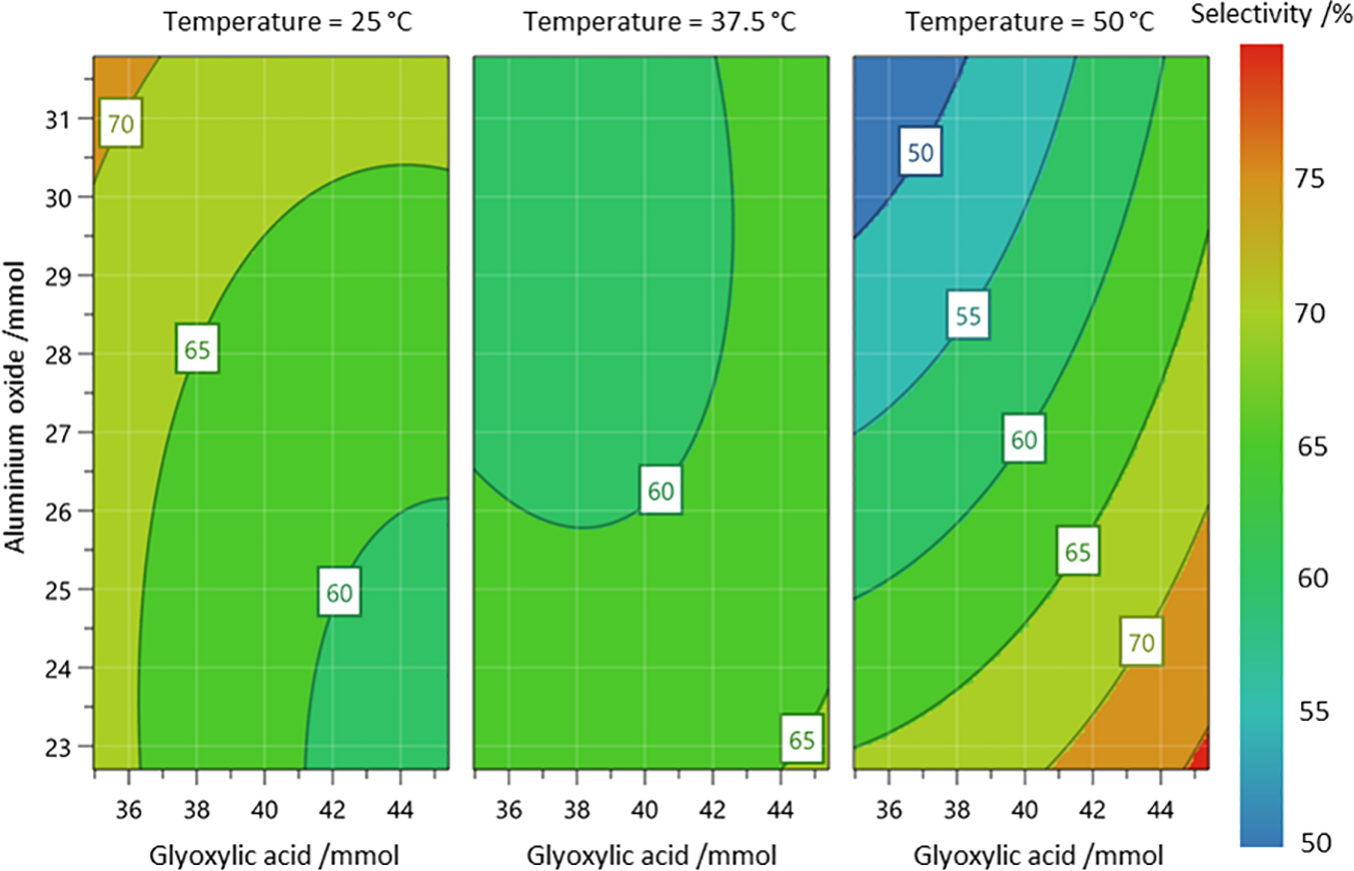
Contour plot for the selectivity of the reaction forming the desired intermediate, **12**. Data was used from the original publication by Minisci and co-workers to refit the model and plot the response surface using MODDE Pro.^40^

The first DoE study explored the following factors: amount of glyoxylic acid, amount of aluminum oxide, reaction temperature, and amount of sodium hydroxide, while fixing the amount of catechol, volume of water, and reaction time at convenient levels. This design resulted in 18 experiments (2^4^ + 2 “center” experiments). However, it was found that an excess of sodium hydroxide results in much greater rates of impurity formation, hence another study was performed under fixed, less basic conditions. The resulting three factors were therefore inputted into the second full factorial design of nine experiments (2^3^ + 1), where the responses of recovered catechol, selectivity of desired product, and yield of desired product were measured. A statistical model was constructed for each response, and the response surface for the selectivity of the desired intermediate, **12**, is plotted in Figure 3. Using this information, it was determined that to achieve an optimum product output, the amount of glyoxylic acid must be increased, the quantity fraction of catechol:aluminum oxide must fall within the range of 2.17–2.28, and the temperature must also be increased. After further experiments using this information at higher factor bounds, the selectivity of the process was improved to 90.5% with a conversion of 78.4%, where the unconverted catechol could be easily recovered and recycled.

**Figure 4:**
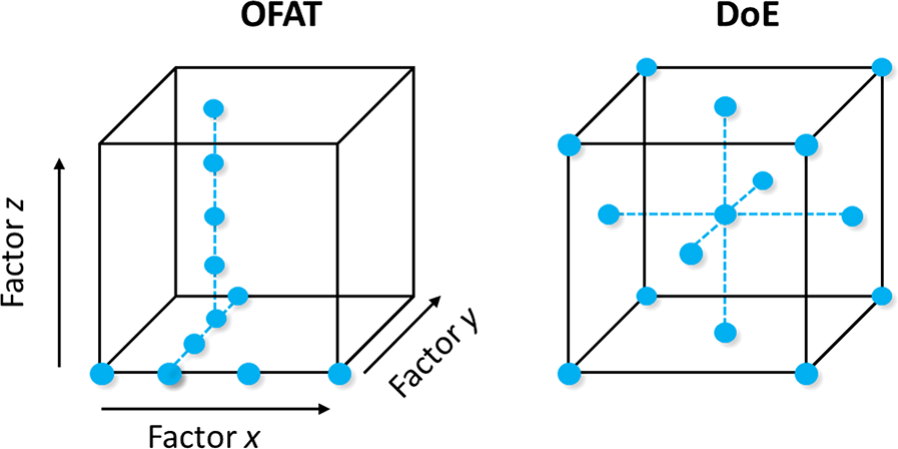
Parameter space exploration expected when comparing a typical OFAT optimization with a DoE design, where • represents an experiment. The DoE shown represents a CCF experimental design. Note that an OFAT optimization does not require a predetermined number of experiments and therefore may or may not exceed the number of experiments in a given DoE design.

There are many advantages to running optimization campaigns using DoE. The use of predefined, space-filling experimental designs removes the necessity for chemical-intuition-guided optimization, and it has been shown numerous times to be a more effective methodology.^23,33^ This space-filling experimentation, as shown in Figure 4 when compared to more conventional OFAT studies, allows statistical models to be constructed to describe the chemical process across the entire parameter space; this is particularly powerful for reaction prediction and allows response contours to be generated.^16^ These statistical models are also often intuitive for a chemist to use because the responses are described as a direct result of the changing factors which can be much easier to interpret than calculating responses using physical models such as kinetic models. These considerations are advantageous in many chemistry situations and are conducive to the efficient optimization of chemical processes.

There are, however, some practical disadvantages to using DoE for optimization that may lead to the necessity of employing other techniques. Although some research groups utilize coding (or packages in particular languages, such as pyDoE) and statistical expertise to perform DoE campaigns,^41,42^ most researchers use paid software packages specifically designed for DoE. These options carry either an expertise or a cost burden (or both), which may have classically hindered the uptake of the technique, particularly for smaller research organizations. However, software options have undoubtedly helped to facilitate the employment of DoE overall due to the high expertise barrier for typical bench scientists to use the statistical methods unaided. Another major disadvantage is the difficulty in exploring categorical variables in DoE studies, as these experimental designs are only suitable for continuous variables. One way to incorporate categorical variables, such as solvent or catalyst, would be to describe them with suitable continuous descriptors that can then be translated to real-world categorical choices; see more information on molecular parametrization in section 6.2.

As DoE builds a statistical model, it has only an empirical meaning rather than physical, therefore, there is no ingrained physicochemical information about the optimized process within the model; this means that model responses are only considered to be accurate within the explored bounds of the experimental factors.^43,44^ For example, if the reaction time is explored as a factor as part of a DoE study between the bounds of 5–30 min, extrapolating the model to predict responses after 60 min would likely result in inaccuracies, and further study must be conducted to predict these outputs. Furthermore, the number of experiments that are required to be performed in parallel for some DoE studies may be large, depending on the amount of reaction material or time required to conduct these experiments, this may be prohibitive in some circumstances.^45^ However, the rise of highly automated experimental platforms with online analytics provides a powerful option for chemical process development in many cases by miniaturizing and automating experimentation using DoE.

DoE has been used extensively in the optimization of chemical processes, particularly in pharmaceutical and fine chemical settings. DoE is often used for studies relating to enhancing the yield^46−57^ and purity^58−62^ of particular products but is also used for drug formulations^63−68^ and delivery,^69−71^ analytical method development,^72−75^ and more.^76−79^ This is because there are numerous and undisputed benefits to the use of DoE for experimental parameter screening and optimization, especially when compared with traditional human intuition-guided experimentation. With the rise of user-friendly software packages and the increased awareness of the chemical community, there has been a large uptake of this statistical method in recent years, although the technique itself has been around since the mid-20th century.^34^ As the advantages of DoE are harnessed, and more industrial job roles will require familiarity with the technique,^35,80^ an organic evolution of academic departments will also occur where there is more teaching of the topic and utilization of the methods for optimization practices. It is therefore a possibility in the future that DoE optimization becomes routine, regardless of the research setting, where the modernized laboratory contains enabling equipment for these studies, with chemists possessing highly diversified skillsets.

### 3.1 Statistical Modeling

A mathematical, empirical model is featured at the center of each DoE study, built from real-world chemical experiments that relate experimental factors to chemical responses. The general format for the model involves fitting coefficients for each experimental factor, as well as for each 2-factor-combination possible. Therefore, the model for a 2-factor experiment is shown in eq 1, where *x*_1_ is variable 1 (for example, temperature), *x*_2_ is variable 2 (for example, reaction time), *y* is the experimental response (such as reaction yield) and *b*_*n*_ is the coefficient term for the variable(s) of interest (determined by regression). Therefore, by replacing *x*_1_ and *x*_1_ with actual values for the factors considered, it is possible to predict the response for any experiment, including experiments with reaction conditions that have not actually been conducted. As any point in the parameter space can be predicted this way, the entire space can also be represented graphically as a contour plot; this allows the behavior of the factors to be more easily understood and interpreted, as well as allowing predicted response maxima to be easily identified. The response is often also mathematically transformed to give better predictability between two bounds, e.g., log 10 transformed so that a yield prediction cannot exceed 100%.
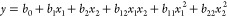
1

As described in the empirical model, it is not only the experimental factors that have an effect on the response but also the interactions between these factors; this is an attribute of DoE that is difficult to reproduce through methodologies that utilize human chemical intuition.^81,82^ These interaction terms within the model indicate how the experimental factors influence reaction output, when other factors are also changed alongside them. When considering the optimization of some generic reaction while exploring the factors of reagent equivalents and temperature, it may be a significant factor in the modeling of the data to include an interaction term between these factors. In real terms, this interaction could indicate that a higher temperature has a positive influence on the reaction output only at higher reagent equivalents. Similarly, these factors may have an interaction term with themselves, as described by a squared term. For example, a squared temperature model term could indicate that temperature has a larger effect on the response at elevated temperatures, meaning that temperature has a nonlinear effect on the reaction within the explored parameter space. These interaction considerations can typically give a better description of the experimental data, as all synergistic effects between the factors are incorporated into the model. However, all experimental factor and interaction terms can be added or removed from a DoE model depending on whether their contribution to the model is significant.

To accurately isolate and determine interaction effects, specific DoE designs can be implemented. Within each design, the experimental factors are split into respective levels: these denote the degree of the experimental factor and are conventionally labeled between the minimum (−1) and maximum (+1). For example, a three-level design that is exploring reaction temperature (10–50 °C) would use the levels −1, 0, and 1, which correspond to 10, 30, and 50 °C, respectively. These levels are defined to ensure that the entire parameter space can be explored regardless of the factor range. Each experimental design may explore different levels, which also depend on the number of factors to be explored, in order to identify specific interaction effects and remove confounding (uncertainty).^83,84^ Depending on the practicality of running experiments, it may be necessary to balance model accuracy with experimental measurements, as some designs that feature several factors may require many more experiments at different levels for a marginal increase in predictive power. For example, to estimate all terms for a model containing five experimental factors, a three-level full factorial design would require 246 experiments, while a face-centered central composite design would only require 29 with a minimal reduction in predictive accuracy. It is therefore important for the bench scientist to identify the optimal experimental design for their purposes to avoid conducting unnecessary experimental observations, leading to additional time and material costs. For more detailed information on DoE designs, also refer to Kumar and co-workers.^35^

### 3.2 Conclusions

Chemical reaction optimization using design of experiments can be very powerful when attempting to identify regions of optimal parameter space. The methodology has a relatively low expertise barrier-to-entry, given the advent of DoE software, and provides bench scientists with tools to identify significant experimental variables and model their data.^34^ Although there are several options for DoE designs, the statistical knowledge necessary for chemists to select the correct experimental procedure (based on their needs for a given experimental outcome) and analyze the resulting data is low. Because of the relative ease of the technique, DoE can be easily taught to chemistry students and adopted for use in academic laboratories as intuition-based methods are superseded.^1,85,86^ Furthermore, the ubiquity of DoE in process laboratories in industry highlights that these statistical methodologies must be taught to chemists; this will help to develop the skillsets of the students and familiarize them with common optimization protocols that they are likely to encounter in future. For further detailed reading on statistical modeling within DoE, refer also to Telford^87^ and Severin and co-workers.^88^

## 4 Kinetic Modeling

The use of kinetic modeling, featuring a mechanistic model rather than a statistical one, is also common for reaction understanding and optimization, especially in process laboratories in industry and academia. Kinetic models are constructed from a scientific understanding of the chemical process^89,90^ rather than statistical relationships between experimental factors and outcomes. In contrast to DoE, undergraduate courses typically cover kinetic analysis in detail as part of their core physical chemistry modules, covering theories on collision, rate laws, and some basic physical-organic concepts. However, the more practical uses for kinetic analysis (reaction optimization, mechanism elucidation, etc.) are seldom explored by many chemists and are typically reserved for chemical and process engineers. This could be because of large expertise gaps experienced by chemists, or simply because they are unaware of the benefits of kinetic studies for their processes. Because of this distinct knowledge gap, practical kinetic analysis is discussed in this review and how it relates to reaction optimization. When these kinetic models are constructed, they enable scientists to understand and simulate reactions to determine optimal regions of parameter space in silico.

The physical modeling of a reaction typically features the rate laws of each individual chemical step and their corresponding rate constants. The main assumption when using a physical model is that the reaction kinetics follow the law of mass action; this states that the rate of an elementary reaction is directly proportional to the product of the concentrations of the reactants, raised to the power of their stoichiometric coefficients.^91^ Written simply, this relates the order of a chemical species within an elementary step to the number of molecules reacting within that step. For the reaction shown in eq 2 (where *x*_1–3_ are chemical species and α/β/γ are their stoichiometric coefficients), based on the law of mass action, the reaction rate can be described by eq 3 and therefore eq 4. The kinetic rate constant, *k*, therefore determines the speed of the reaction as well as the reactant concentrations.
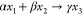
2
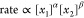
3
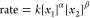
4

The law of mass action is applicable in almost all cases and is typically only inappropriate when concentrations of particular substrates are very low. More commonly encountered are very fast elementary reactions that occur in some processes, where it is more appropriate to describe an entire reaction with an observed rate rather than the combination of its individual elementary parts. This leads to circumstances where chemical species that are reported to have a zero-order, second-order or even noninteger-order dependence, as it is much more practical to describe the physical model in this way.^92−97^

**Scheme 4:**
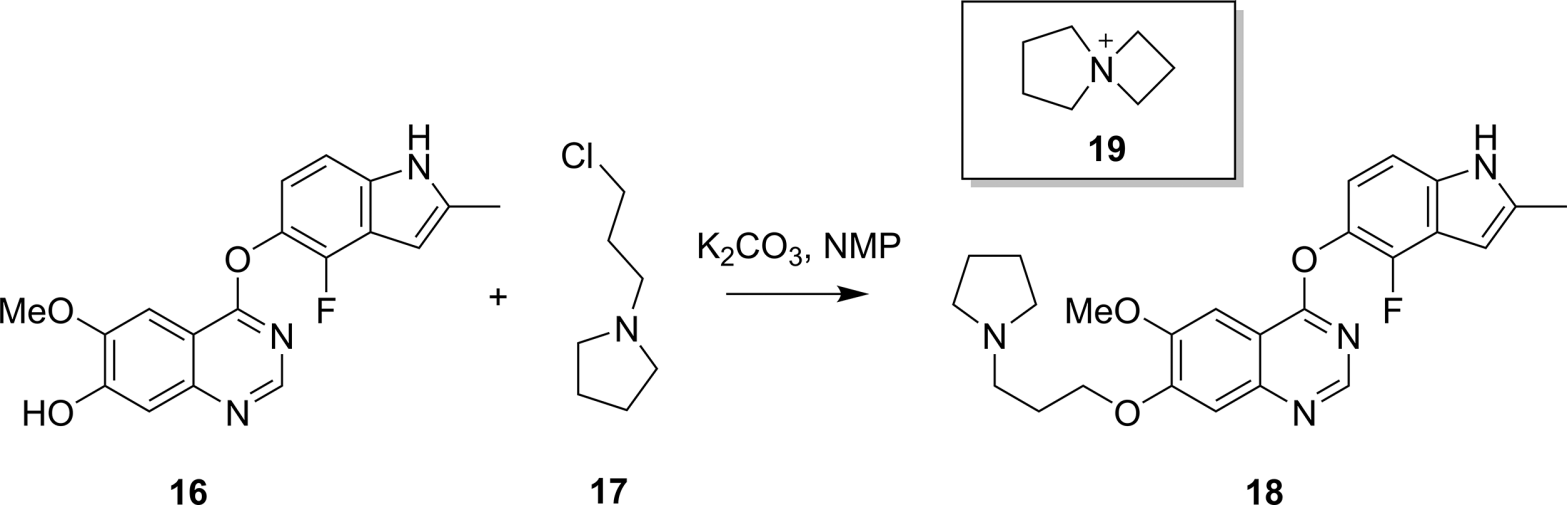
Alkylation of the Indolphenol, **16**, with the Chloropyrrolidine, **17**, to Form the Desired Cediranib Product, **18** This reaction was found to proceed via the azetidinium intermediate, **19**, as a result of kinetic modelling.^100^

The physical models generated from kinetic analysis contain ingrained chemical information that, unlike their empirical model counterparts, can be used to extrapolate reaction predictions outside of previously conducted experimental constraints.^98,99^ The determination of reaction orders and rate laws within a model is used to optimize outputs but also to increase overall chemical understanding. As a result of impurity formation upon scale-up, Ashworth and co-workers studied the kinetics of the alkylation of the indolphenol, **16**, with the chloropyrrolidine, **17**, to form cediranib, **18**, which is a pharmaceutical treatment for solid tumors (Scheme 4).^100^ The authors found that overall second-order kinetics, consistent with a direct nucleophilic substitution between the anion of **16** and **17**, were not observed in their experiments. Instead, overall first-order kinetics suggested an initial slow step to form another species. Further experimentation confirmed that **17** slowly reacted to form the azetidinium ion, **19**, which then reacted quickly with **16** to obtain the desired product. This mechanistic understanding was achieved as a direct result of kinetic experiments, which led to further optimization of the overall process (solvent selection and base equivalents) for an increase in overall reaction yield.

**Scheme 5:**
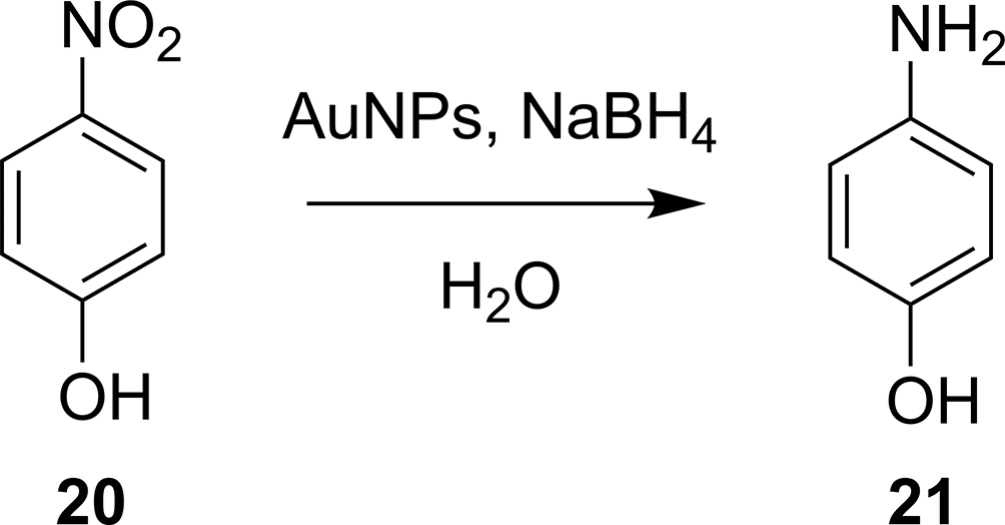
Aqueous Reduction of 4-Nitrophenol, **20**, to 4-Aminophenol, **21**, Using Gold Nanoparticles (AuNPs) and NaBH_4_^101^

**Figure 5:**
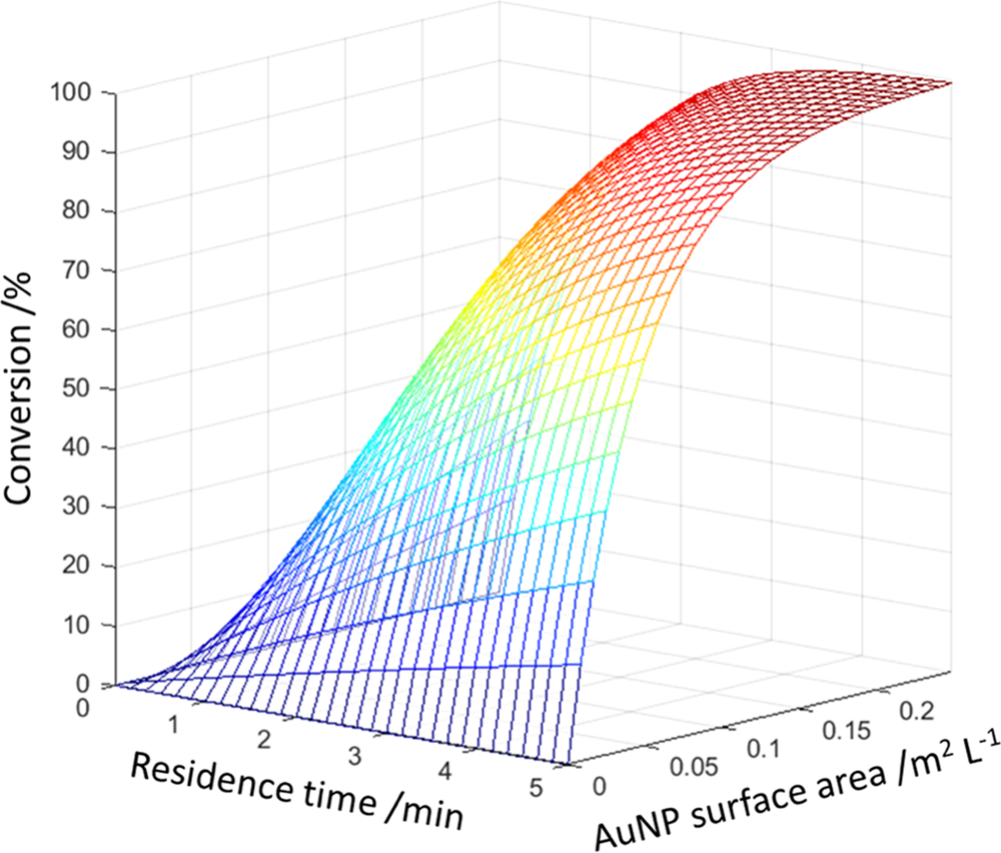
Kinetics-derived response surface for the conversion of 4-nitrophenol, **20**, to 4-aminophenol, **21**, when exploring the variables of residence time and AuNP surface area per liter.^101^

As reaction progression can be described by the rate laws in eq 4, predictions of reactant/product concentrations can be made for any collection of reaction variables such as reaction time, reagent equivalents, and temperature. This allows response surfaces to be plotted in the same manner as with DoE studies, allowing the visual determination of high-interest experimental parameters for process optimization. One example of this application is in the continuous-flow aqueous reduction of 4-nitrophenol, **20**, to 4-aminophenol, **21**, using gold nanoparticles (AuNPs) by Chamberlain and co-workers (Scheme 5).^101^ This kinetic study related the surface area of AuNPs and flow residence time to reaction conversion, highlighting the optimal reaction conditions for the scale-up of the pharmaceutical building block, 4-aminophenol, as shown in Figure 5.

### 4.1 Conventional Approaches

**Figure 6:**
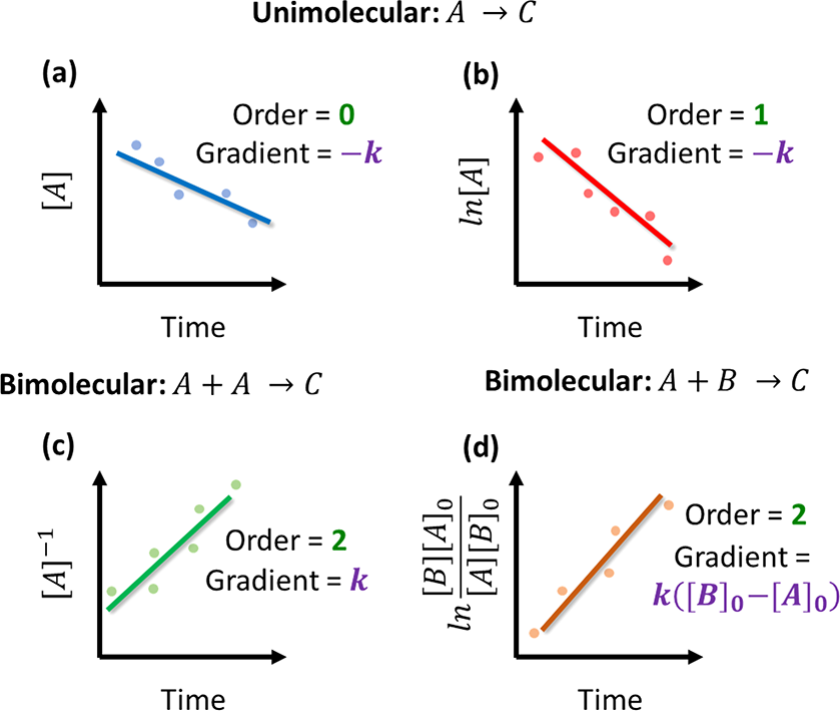
Common conventional kinetic analysis techniques for the determination of: (a) unimolecular zero-order kinetics, (b) unimolecular first-order kinetics, (c) bimolecular second-order kinetics between the same reactants, and (d) bimolecular second-order kinetics between different reactants.

When fitting a physical model of rate laws to a chemical process, the reaction order and rate constants must be experimentally determined. For a mechanistic model that incorporates reaction temperature, activation energies must also be identified. Conventionally, mathematical transformations to concentration–time data are applied to identify both the reaction order and rate constants in one plot; this can then be repeated at multiple temperatures to obtain the activation energies for the reaction.^102−106^Figure 6 shows the most common, classical data transformations to obtain rate law information from kinetic experiments.^107^ For each case, a linear fit to the transformed data indicates that the reaction order is correct, while the gradient of the fit gives information on the rate constant. For a unimolecular reaction, a zero-order reaction is confirmed with a linear fit to the concentration–time data (a) while a first-order reaction can be confirmed via a log-transformed plot of the data (b). For bimolecular reactions, if both reactants are the same, then an overall second-order reaction can be confirmed simply from the plotting of the reciprocal of the concentration data (c). However, when both reactants are not the same, more complex plotting can confirm an overall second-order reaction (d). Additional modeling, such as the conventional modeling of enzymatic (and sometimes other catalytic) reactions, known as Michaelis–Menten kinetics, can also be applied; for more information refer to a recent review by Johnson.^108^

These techniques are still employed regularly today in both teaching and research laboratories. For example, a study by Etua and co-workers^109^ confirmed second-order reaction kinetics of a benzaldehyde oxidation, and work by Guégan and co-workers^110^ confirmed first-order reaction kinetics in an epoxybutane polymerization; both cases are recent examples of how these conventional methodologies are still used. As can be seen from Figure 6d, the mathematics required to extract kinetic information grows much more complex as the reaction deviates further from very simple chemistry. Therefore, these conventional approaches become less appropriate when studying processes that are multistep, contain multiple reaction pathways, and have impurity formation as the mathematics becomes more inaccessible. In these complex cases, it is more common to use modern kinetic analysis techniques depending on the chemistry, such as reaction progress kinetic analysis,^111^ or kinetic fitting software, such as Compunetics.^101,112^

### 4.2 Modern Techniques

**Scheme 6:**
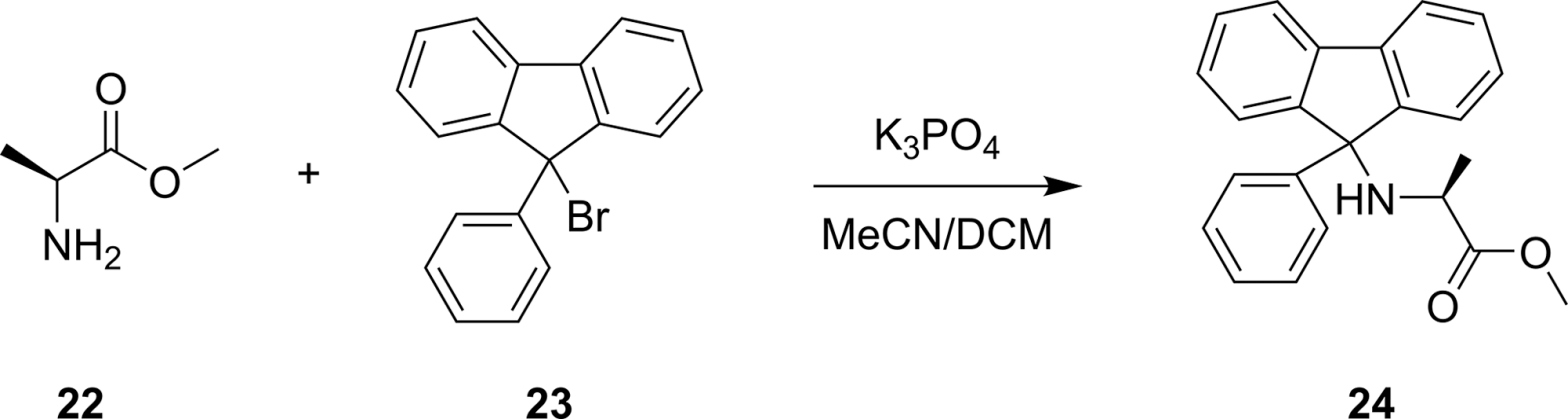
Reaction of Al-Me, **22**, with PfBr, **23**, to Form the Protected Amino Acid Pf-Al-Me, **24**(92)

**Figure 7:**
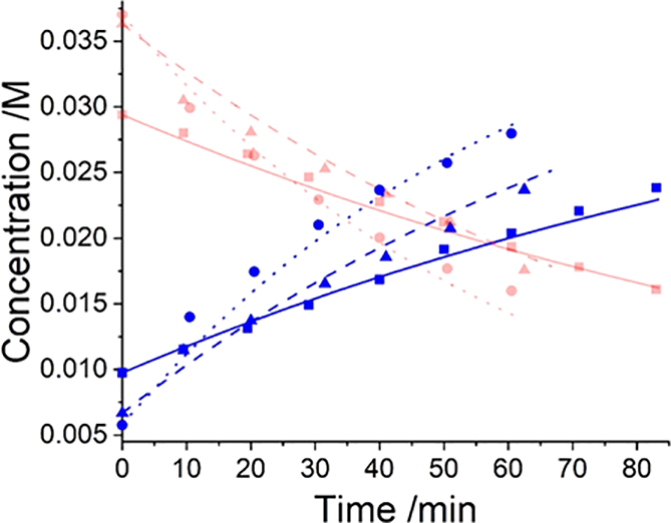
Kinetic profiles for three kinetic experiments at 30 °C, 35 and 40 °C, where red plots indicate PfBr concentrations and blue plots indicate Pf-Al-Me concentrations. At 30 °C: blue solid squares = experimental data, **—** = kinetic fit. At 35 °C: blue solid triangles = experimental data, **- - -** = kinetic fit. At 40 °C: blue solid circles = experimental data, _**······**_ = kinetic fit.^92^

In every chemical case, regardless of the complexity of the process, it is possible to write coded solutions for kinetic analysis using rate constant solvers (using differential equation counterparts to eq 4).^113−115^ Although this is a useful strategy that is often employed by engineers,^116−120^ it is rarely conducted by chemists, as there is a high expertise barrier to access this form of kinetic analysis, namely, coding and mathematical knowledge. Software solutions have been developed to aid in this kinetic model fitting (Compunetics,^121^ Berkeley Madonna,^122^ DynoChem,^123^ COPASI^124^) that often require minimal coding expertise, which have been adopted by process chemists but have still had relatively low uptake within the wider chemistry community. One example of fitting a kinetic model using rate constant solvers was shown in our lab for the determination of the overall first-order reaction of alanine methyl-ester (Al-Me), **22**, and 9-bromo-9-phenylfluorene (PfBr), **23**, to form the protected amino acid (Pf-Al-Me), **24**, as shown in Scheme 6.^92^ This approach computationally identified the “best-fit” parameters for the rate constants and activation energies, with the fit to the experimental data shown in the combined plot in Figure 7.

Another common modern kinetic analysis technique used for the determination of physical models, particularly for catalytic processes, is reaction progress kinetic analysis (RPKA).^111^ This methodology was pioneered by Blackmond and represents a systematic experimental procedure for kinetic analysis through sequential experimental/analytical steps involving reaction rate/time data. Rather than coding kinetic fitting solutions, this methodology features various plotting techniques and uses qualitative visual confirmations of overlaying graphs to determine catalyst/reactant orders, catalyst deactivation, and product inhibition. Therefore, precise kinetic parameters cannot be elucidated, but the plots required are simple to construct and easy to interpret, which allows easy determination of this kinetic information. Because of its systematic approach to analysis, RPKA has been widely adopted in process chemistry settings and reported in several applications.^125−130^

**Scheme 7:**
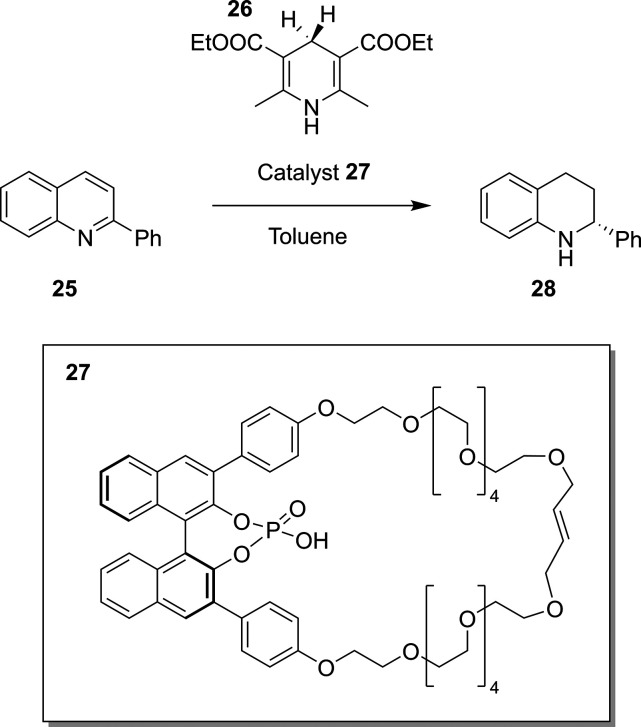
Stereoselective Reduction of 2-Phenylquinoline, **25**, to Yield the Tetrahydroquinoline, **28**, Using the Hantzsch Ester, **26**, and the Macrocyclic Catalyst, **27**(131)

One recent example by Niemeyer and co-workers used RPKA in the regio- and stereoselective reduction of 2-phenylquinoline, **25**, with the Hantzsch ester, **26**, using the macrocyclic catalyst, **27**, to yield the tetrahydroquinoline, **28**, as shown in Scheme 7.^131^ This work highlighted, using the RPKA methodology, that the process was first-order with respect to both substrates and the catalyst, and that there was no observed catalyst deactivation or product inhibition over time. Each piece of information gained from this study can thereby help in further process development, both for reaction condition optimization and scale-up suitability.

Variable time normalization analysis (VTNA) is another technique that, alongside RPKA, falls into the dubbed category of “visual kinetic analysis”.^132^ VTNA can obtain the same chemical and physical model information as RPKA but does not require rate/time data; instead, VTNA can be used directly with concentration/time data, which requires fewer experiments to obtain and less data manipulation.^133,134^ As VTNA requires only simple graphical transformations with easily obtained data, this methodology has also been reported in many process chemistry applications and chemical optimization studies.^135−138^

**Scheme 8:**
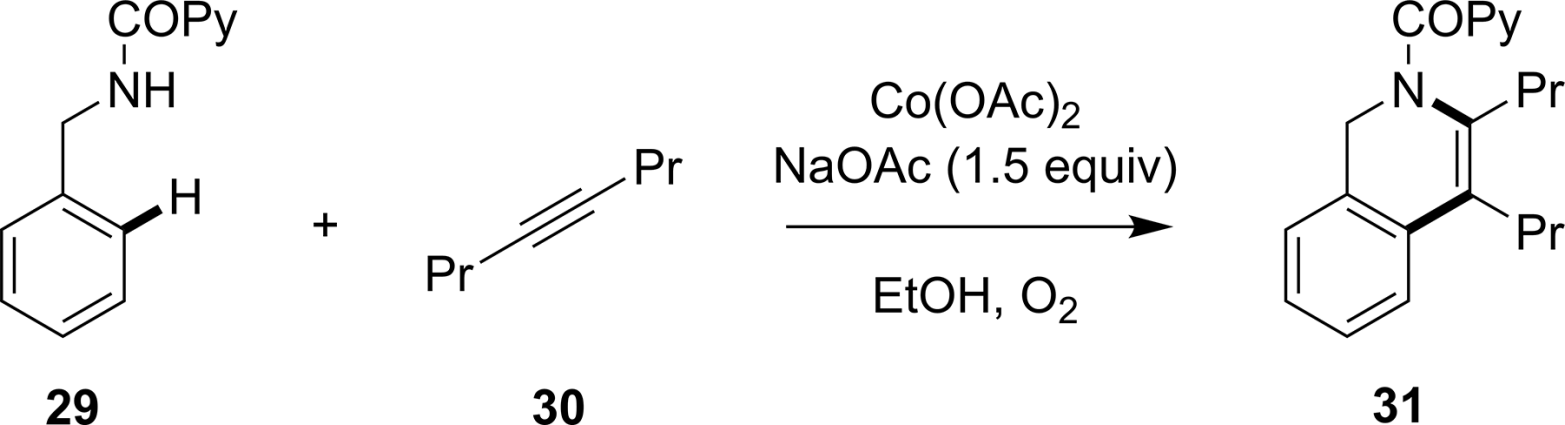
Cobalt-Catalyzed C–H Functionalization/Alkyne Annulation Reaction of **29** with **30** to Form the Dihydroisoquinoline Product, **31**(139)

One recent example by Carretero and co-workers showed how VTNA was utilized in the cobalt-catalyzed C–H functionalization of *N*-benzylpicolinamide, **29**, with 4-octyne, **30**, to yield the dihydroisoquinoline product, **31**, as shown in Scheme 8.^139^ This study showed that the reaction exhibited a first-order dependence on the cobalt concentration, a zero-order dependence on the alkyne and a partial negative-order in the benzylamide concentration. The authors suggested that this partial negative-order finding was a result of off-cycle unproductive binding interactions with the catalyst, thereby decreasing the effective concentration of catalyst available. Therefore, for further optimization work and scale-up, this quantification of reaction orders has been crucial in showing that there must be an optimal benzylamide concentration range whereby productivity is maximized.

### 4.3 Outlook

Kinetic modeling is a very powerful tool for the optimization of chemical processes and represents a much more systematic approach than traditional OFAT optimization. The mechanistic models used in kinetic analysis also provide chemical insights and scientific understanding that DoE does not but may also be more difficult to interpret and conduct experimentally as time-series data is paramount. Although software solutions have been created to lower the expertise barrier for kinetic analysis, the uptake of kinetic modeling techniques is still relatively low among chemistry researchers as the analysis is typically assigned to physical-organic or engineering colleagues, often unnecessarily as many reactions are simplistic and easy to fit a physical model to. Visual kinetic analysis, although a relatively new methodology, has helped to provide an accessible framework for chemists to obtain semiquantitative model information from their processes without the need for coding or software. More complex techniques that feature parameter estimation for kinetic modeling have not been covered, but interested readers are directed to reports on model-based design of experiments (MBDoE).^140−142^ As robust physical models are often useful for optimization and necessary for scale-up, familiarity with these modeling techniques is very important as our laboratories become more interdisciplinary and connected.

## 5 Self-Optimization

Self-optimization is a modern approach to automating the discovery of optimal reaction conditions for chemical processes which does not require the determination of explicit mechanistic or empirical models. Self-optimization proceeds through iterative cycles of automated reaction execution, quantification, and algorithmic condition selection to efficiently identify optimal reaction conditions to maximize process metrics (yield, selectivity, etc.). Although self-optimization was initially applied to tuning analytical instruments as early as the 1970s,^143^ DeMello and co-workers^144^ first introduced the concept of self-optimization of chemical reactions in 2007, which led to further adoption by many other research groups in the following years. DeMello and co-workers focused on the synthesis of CdSe quantum dots, but subsequent works have applied self-optimization to a wide range of synthetic organic reactions including oxidation,^145^ Diels–Alder,^146^ methylation,^147^ Paal–Knorr,^148^ Suzuki–Miyaura cross-coupling,^149,150^ hydrolysis,^151^ and C–H activation.^152^

**Figure 8:**
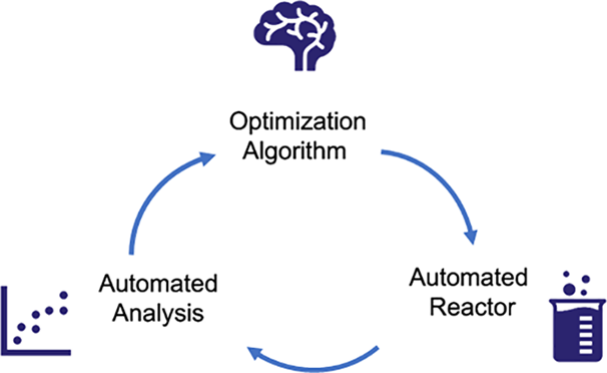
Three parts of a self-optimizing reactor are an automated reactor system, an analytical method, and an optimization algorithm.

Self-optimization is often conducted using automated reactors that can independently execute reactions at a specified set of reaction conditions. Automated analytical instruments then quantify the individual components of a reaction mixture, followed by an algorithmic suggestion of new reaction conditions based on previous data to improve key reaction outcomes. Self-optimization research can therefore often be divided into three subsections that map directly on to these three key aspects: development of automated reactors, development of automated analytical methodologies, and development of optimization algorithms (adapted for a specific chemical problem). One or more of these developments are typically reported in individual works in the literature. These ideas are shown conceptually in Figure 8.

The primary goals of self-optimization are to reduce the number of experiments required to optimize a reaction and the experimental burden on the bench scientist, saving time and money. Additionally, self-optimizing systems offer a more consistent way of generating data than human driven reaction optimization. This means that self-optimization has the potential to not only accelerate reaction optimization but also provide data that will enable predictive modeling in the future. Currently, self-optimization has been adopted by both academia and industry, although work from the former has been published more readily.

### 5.1 Automated Reactors for Self-Optimization

**Figure 9:**
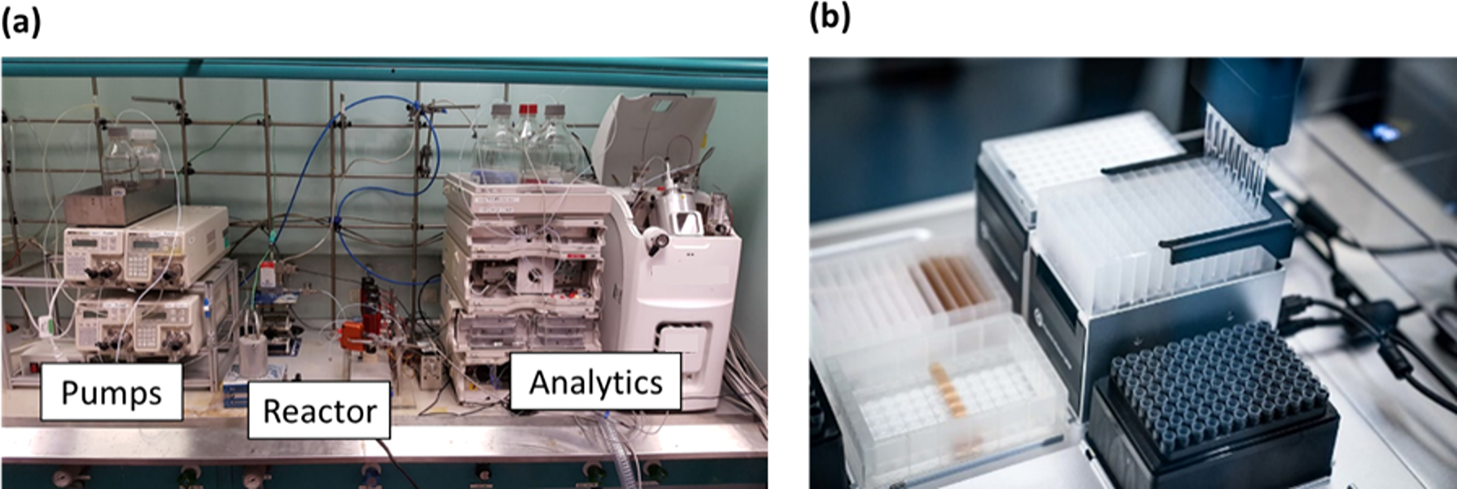
Examples of automated reactors. (a) A bespoke automated flow reactor equipped with pumps, reactors, and analytical equipment.^153^ (b) A commercial robotic liquid handler that can be utilized as an automated batch reactor (see section 6.2 for more details).

Automated reactors must be able to receive and execute a set of reaction conditions without human intervention. Continuous flow and batch reactors have been applied to self-optimization, and prominent examples from the literature are herein discussed, but two example reactor setups are shown in Figure 9.

#### 5.1.1 Automated Flow Reactors

Most self-optimization studies in the literature have employed automated flow reactors.^154,155^ Utilizing developments in continuous flow chemistry, these automated flow reactors use pumps to deliver solutions at a desired flow rate to a temperature-controlled reactor. By pumping solutions of starting materials and reagents at varying flow rates, precise stoichiometries and reaction times can be achieved. Automated flow reactors are attractive because a bespoke system can be quickly assembled using commercially available parts, or a complete system can be purchased from several specialized vendors. Building a bespoke system offers flexibility and lower costs, while complete systems enable faster (and often easier) application deployment.^156^ An additional advantage of automated flow reactors is that the optimal conditions found in an automated flow reactor can be utilized for medium-to-large scale production (grams to kilograms per week) by either running the reactor for an extended period^157^ or numbering-up,^158^ as discussed in section 7.

However, three major challenges are faced by researchers using automated flow reactors for self-optimization. First, automated flow reactors can consume large amounts of starting material and solvent due to the need to flush the reactor when changing conditions. The standard heuristic is to wait at least two residence times (i.e., twice the amount of time necessary for material entering the reactor to exit) prior to initializing analytical measurements, so much of the reaction material is directed to waste. Second, changing categorical reagent conditions such as catalyst, base, or solvent is nontrivial in a standard automated flow reactor because each pump must be loaded with a particular reagent prior to reaction execution.^159^ Third, automated flow reactors suffer from standard issues with flow chemistry, particularly reactor clogging due to precipitation of solids,^160^ and limits in the maximum residence time of compact reactors.^161^

**Figure 10:**
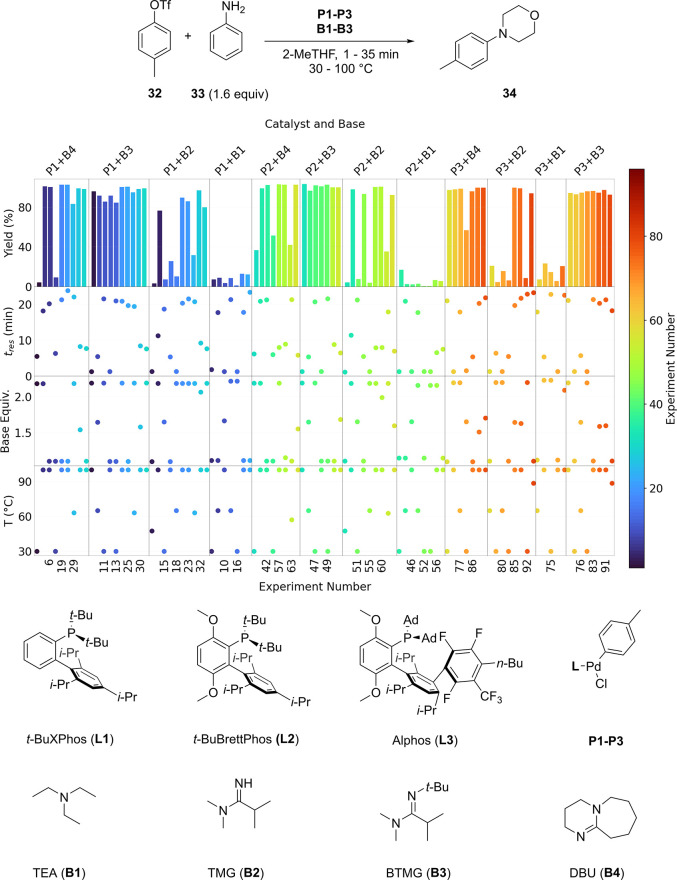
Development of a Buchwald C–N cross coupling between *p-*tolyl triflate (**32**) and aniline (**33**) via self-optimization in an automated droplet flow reactor.^163^ Three continuous variables (residence time, base equivalents, and temperature) and two categorical variables (catalyst and base) were varied to maximize the yield of 4-(*p*-tolyl)morpholine (**34**). In the chart, each column contains data for a different catalyst and base combination, and the experiments in each column are shown left to right in the order they were selected by the optimization algorithm. Additionally, the color bar shows experiment selection order.

To overcome challenges of material consumption and changing reagents in automated flow reactors, researchers have developed automated droplet flow reactors.^162^ These reactors employ liquid handlers that transfer the individual components for a reaction into a sample loop prior to injecting them as a droplet into the reactor tubing. The droplets can be as small as several hundred microliters, resulting in large reaction material savings when compared with traditional continuous flow experimentation. For example, Jensen and co-workers demonstrated the self-optimization of a C–N cross-coupling reaction in an automated droplet flow reactor varying catalyst and base with a liquid handler (see Figure 10); their reactor consumed less than 200 mg of starting material.^163^

A variety of technologies have been developed to address other standard issues with flow chemistry. Methods to prevent clogging include continuous stirred tank reactors (CSTRs) that can facilitate slurries^164^ and reactors with baffles to improve mixing and reduce precipitation.^165^ To overcome challenges with limited residence time in flow reactors, researchers have applied oscillatory droplet flow reactors that move droplets back and forth inside a fixed length tubing until the desired reaction time is achieved.^166−168^ These oscillatory systems could, in theory, enable very long reaction times (hours), although in practice they have only been used for reactions with shorter reaction times (minutes).

Overall, the field of flow chemistry offers a powerful set of tools for building automated reactors for self-optimization. By either building or buying automated flow reactors, research groups and industry can quickly access the basic laboratory tools needed for building self-optimizing systems. However, particular care must be taken for solid handling and the screening of categorical parameters (such as solvent, catalyst etc.) as vital modifications to existing equipment may be necessary.

#### 5.1.2 Automated Batch Reactors

**Figure 11:**
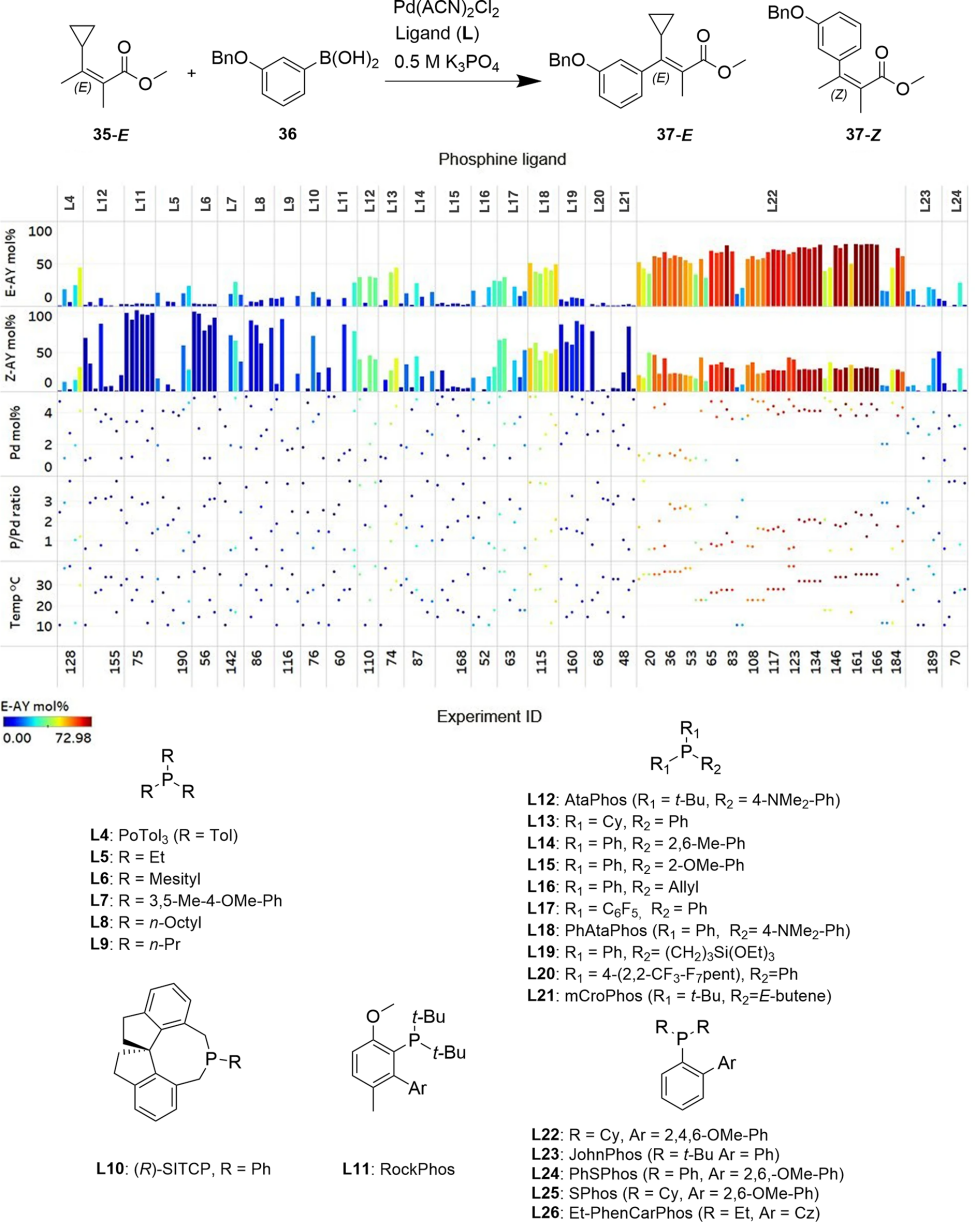
Development of a stereoselective Suzuki coupling between sulfonate **35**-*E* and boronic acid **36** to form **37**-*E* and **37**-*Z* via self-optimization in an automated batch reactor. In 161 experiments, the yield of **37**-*E* was improved from 30% nominal to 70%, and the *E*/*Z* ratio from 1.5:1 to 2.5:1. AY represents assay yield.^149^

Recent work has shown that automated liquid handling robots, historically deployed for high-throughput screening (HTS) in biological applications, can also be used in combination with high-throughput screening microplates to create automated batch reactors for self-optimization.^15,149^ Using these liquid handling instruments, it is possible to screen many categorical variables, such as catalysts, ligands and solvents. Hein and co-workers conducted self-optimization of a stereoselective Suzuki coupling reaction using a ChemSpeed liquid handling robot, varying the ligand, several stoichiometries, and temperature to maximize the formation of the *E*-product and minimize the *Z*-product, as shown in Figure 11.^149^ This self-optimization campaign led to more than a 2-fold increase in yield of the *E*-product (30% nominal to 73% optimized) and a notable increase in the *E*/*Z* ratio (1.5:1 nominal to 2.5:1 optimized) within 161 experiments.

As automated liquid handling robots are common among high-throughput experimentation (HTE) and process development groups in industry, automated batch reactors represent a significant opportunity for industrial adoption of self-optimization. Indeed, the few publicly available self-optimization studies that have been executed using automated batch reactors were from industrial-affiliated groups. HTE is further discussed, in detail, in section 6.1.

#### 5.1.3 Analytical Techniques

The most common analytical techniques used in self-optimization are chromatographic, either high performance liquid chromatography (HPLC) or ultrahigh performance liquid chromatography (UHPLC).^154^ This trend is likely due to the ubiquity of HPLC and UHPLC instruments in chemical synthesis laboratories and the relative ease with which they can give quantitative data and be integrated into self-optimization systems. For automated flow reactors, HPLC sampling is achieved using a switching valve located at the outlet of the reactor, which can alternate between directing reaction material to waste during changes between conditions and sending aliquots to the HPLC instrument for analysis; this is referred to as online sampling, as material is removed from the flow path for analysis.^169^ This sampling is typically initialized based on the residence time of the reaction or, in the case of droplet flow reactors, an in-line UV cell. In addition to HPLC, gas chromatography (GC) has also been reported in the literature, but this is much less common.^152^

In addition to online HPLC, there have been many reports on developing in-line analytical techniques, such as NMR, IR, UV, and FTIR. In-line NMR has been used to identify both known and novel products synthesized in self-optimizing reactors.^170−172^ Additionally, in-line IR and FTIR has been used independently and in combination with NMR and HPLC for reaction quantification.^170^ The advantage of in-line analytical techniques is they offer fast data feedback, enabling more rapid optimization. However, because the analysis is conducted on the crude reaction mixture, these complex mixtures can suffer from overlapping peaks which often makes quantification difficult. The supporting information of the review by Rincon and co-workers has a list of flow chemistry self-optimization studies and the on-line/in-line analytical techniques used.^154^

### 5.2 Optimization Algorithms

In self-optimization, an efficient optimization algorithm is required to select new reaction conditions based on previous results. Chemical reactions can be viewed as mathematical functions that receive reaction conditions as input values and output reaction outcomes (e.g., product yield, selectivity, etc.).^173^ This functional view of chemical reactions makes it clear why optimization algorithms, which find the optimal values of mathematical functions, can be used to optimize chemical processes. Optimization algorithms iteratively evaluate the output of the function at different input values until a maximum or, if desired, a minimum, is reached. In the case of chemical processes, these iterative evaluations correspond with intelligently suggested experiments to execute in the laboratory until a set of reaction conditions are achieved that give the optimal desired output.

#### 5.2.1 Local Optimization vs Global Optimization

**Figure 12:**
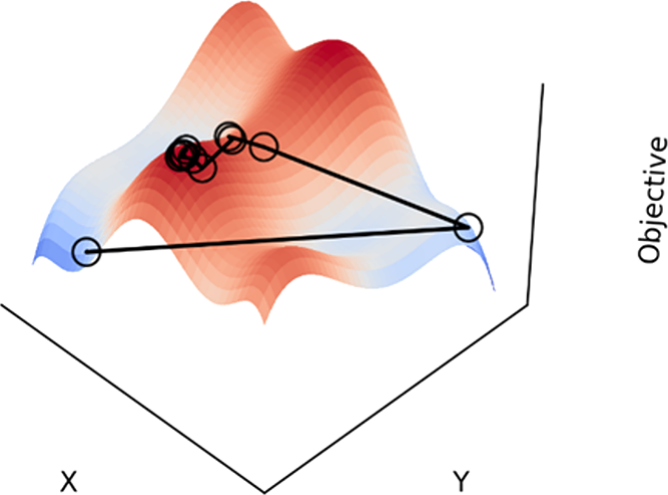
Example of a local optimization algorithm failing to find the global maximum of a function with multiple local optima, i.e., the algorithm finds a maximum peak but not the highest peak. X and Y are hypothetical experimental variables (e.g., temperature, reaction time), and the objective is the value that must be maximized (e.g., yield). Unfilled circles are function evaluations (i.e., experiments). Red indicates local maxima function value, while blue indicates local minima.

The two main classes of optimization algorithms are local and global optimization algorithms. Local optimization algorithms are designed to find the optimal values of a function closest to an initial guess. Therefore, if there is one optimal value, local optimization algorithms will likely find it, but if there are multiple optima, the success of a local optimization method is highly dependent on the initial guess. Example chemical applications of local optimization algorithms include the steepest descent algorithm, which chooses reaction conditions based on the most favorable gradient (direction) in design space to explore,^145^ and the simplex algorithm, which explores the design space based on geometric transformations to exploit perceived favorable areas.^143,147^ The challenge with local optimization algorithms is their dependence on the reaction conditions used to initialize the algorithm; if there are multiple regions of chemical space with local optima, the algorithm could potentially fail to find the best overall reaction conditions for the transformation, as illustrated in Figure 12.

Global optimization algorithms can identify the best value of a function independent of the initial guess but may require more experiments to obtain. Krishnadasan and several other researchers were the first to apply global optimization to self-optimization.^47,144,145^ They used the Stable Noisy Branch and Fit (SNOBFIT), which, as its name suggests, relies on sequential branching and fitting steps. The algorithm begins by subdividing the optimization domain into boxes with one data point each (i.e., branching) and subsequently builds full quadratic models for each box and its nearest neighbors (i.e., fitting).^174^ SNOBFIT achieves global optimization by sampling evenly from the complete reaction condition input space. This has been shown to result in a larger number of experiments than local optimization in exchange for a higher likelihood of finding the optimum for difficult problems (i.e., challenging nonlinear optimization tasks).^145^ SNOBFIT also allows researchers to conduct experiments in batches (i.e., the algorithm makes multiple experimental requests at one time).

More recently, researchers have started to apply Bayesian optimization within chemistry optimization problems.^15,175−178^ This class of optimization algorithm is a subset of Bayesian statistics, which uses probability to express certainty in future outcomes based on past observations. In the case of reaction optimization, when an experimental iteration is completed, a probabilistic model is trained to predict its reaction outcome (e.g., yield) given the reaction conditions. Then, the Bayesian optimization algorithm chooses experiments that balance further exploration of chemical space and exploitation of the known best performing conditions.^179^ Bayesian optimization offers a principled and efficient way to apply global optimization to chemical reactions that is more intuitive and often more effective than other global optimizers.

#### 5.2.2 Categorical Variable Optimization

Each of the aforementioned algorithms only utilize continuous input variables (e.g., temperature, concentration, residence time), but chemistry problems often have categorical variables also (e.g., solvent, ligand, etc.). To address this algorithmic limitation, Jensen presented a branch-and-fit algorithm that eliminates possible values for categorical variables (e.g., particular solvents, ligands, etc.) that lead to poor reaction performance.^150,159,180,181^ The algorithm fits predefined mathematical models for each combination of categorical variables and, once the best categorical combination has been identified, it suggests further experiments to improve the fit of the model. However, this approach requires users to specify a kinetic model a priori, which may be difficult when a full reaction mechanism is not known, as discussed in section 4. Furthermore, because no model is built to describe the relationship between categorical variables, insights cannot be easily drawn about the relationship between different catalysts and bases. This could explain the poor performance seen when this algorithm was used in the aforementioned example of C–N cross coupling (see Figure 10).

Alternatively, it is possible to use optimization algorithms that inherently work with categorical variables. In recent studies, Bayesian optimization algorithms were adapted to automatically learn the relationship between categorical variables from experimental data.^177,178^ These algorithms tended to perform slightly better in finding optimal reaction conditions than the aforementioned strategies that could not learn a relationship between categorical variables and may be a large research area of interest in future. Categorical variables can be explored more easily by quantifying them with various “continuous variable” chemical descriptors, as highlighted in section 6.2. However, more recently, work by Bourne and co-workers^177,182^ showed the effectiveness of their mixed variable multiobjective optimization (MVMOO) algorithm in optimizing categorical/continuous variables without chemical descriptors. In their respective works, they show the use of novel distance metrics based upon Gower similarities that reduce this necessity in both simulated and experimental case studies.

#### 5.2.3 Multiobjective Optimization

Optimization problems in chemistry often involve trade-offs between multiple competing objectives, such as balancing high process yields with low costs, so optimization algorithms need to be able to consider and weight these objectives to find optimal solutions. For example, Jensen and co-workers optimized catalyst turnover number (ratio of the rate of product formation to catalyst usage) with the constraint that yield must be greater than 90%.^150^ This constraint was implemented to prevent the yield being maximized by simply adding higher loadings of an expensive catalyst. In other cases, researchers have optimized a weighted function of multiple objectives.^144,151,183^ Both of these methods require the scientist to make an a priori judgment about the trade-offs between competing objectives and their respective importance. This prior judgment can be limiting and results in a single optimum point identified, whereas there are likely multiple optimal solutions depending on the weightings of the individual objectives.

An alternative approach to considering these trade-offs is the use of multiobjective optimization algorithms. These algorithms explore the full set of trade-offs between multiple objectives.^175,184^ These algorithms construct a Pareto front, which is defined as a set of points in which an improvement in one objective would result in a detriment to another. By presenting a spectrum of trade-offs, this multiobjective approach allows the scientist to select one optimal point with other softer constraints considered (e.g., budget constraints, manufacturability, downstream separation efficiency, etc.). Due to the need to optimize multiple variables and explore globally, multiobjective Bayesian optimization algorithms can be slower than gradient descent or single objective optimization and require further experimentation.

**Figure 13:**
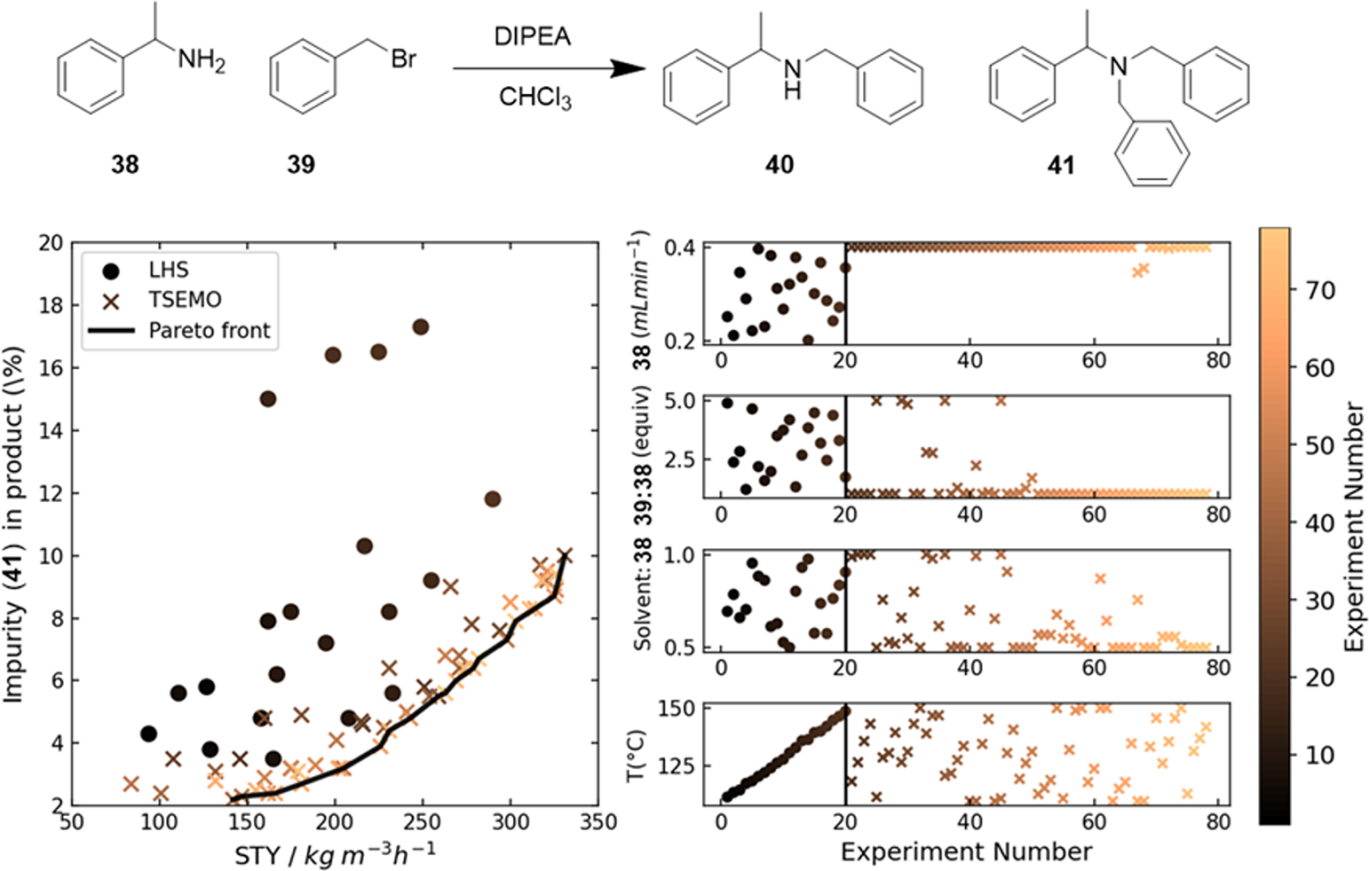
Multiobjective self-optimization of the *N*-benzylation of *N*-benzylation of α-methylbenzylamine **38** with benzyl bromide **39**.^175^ TSEMO^184^ was used to maximize space–time yield of the desired 2° amine **40** and minimize production of the percent impurity of 3° amine **41**. After 20 experiments designed by Latin hypercube sampling (LHS),^185^ TSEMO quickly identified experiments on or near the Pareto front.

Figure 13 shows an example of the multiobjective Bayesian optimization algorithm TSEMO^184^ applied to a *N*-benzylation reaction.^175^ The flow rate of α-methylbenzylamine **38**, ratio of benzyl bromide **39** to **38**, solvent flow rate, and temperature were modified to maximize production of **40**, while minimizing formation of **41**. After 20 experiments designed by Latin hypercube sampling (LHS),^185^ a balanced random sampling technique, TSEMO quickly identified 58 further experimental conditions on or near the Pareto front. The Pareto front indicated that a 60 kg m^–3^ h^–1^ increase in space-time yield (STY) would correspond with an approximate 10% increase in impurity yield, which the scientist can then consider based on the needs of the process.

#### 5.2.4 Benchmarking of Optimization Algorithms

The self-optimization reports explored thus far primarily focus on single experimental case studies, implementing bespoke optimization strategies; this makes it difficult to compare the performance of optimization algorithms objectively. Therefore, recent work has aimed to develop chemically relevant optimization simulations of reactions, or benchmarks, so that these algorithms can be compared without the time and expense of laboratory experiments.^186−188^

Benchmarking rarely finds algorithms that will work in all situations, but they can help filter out poorer-performing algorithms and compare the effect of small changes to algorithms for potential benefits. Furthermore, benchmarking studies can act as postverification of algorithms developed initially on real experiments, as it is possible to run large numbers of repeated simulations to understand the average behavior of these algorithmic techniques.

### 5.3 Future Directions

Self-optimization has the potential to significantly accelerate reaction development by enabling autonomous optimizations of reaction conditions. However, currently, as highlighted by Hein and co-workers, automated reactors often require significant human intervention and adjustment to achieve high quality results.^156^ Therefore, there are still open research questions tackling how to develop highly flexible automated reactors that can adapt to a wide range of chemistry without significant customization. There are also further necessities when conducting reactions that must be addressed, particularly steps that are easy for humans but more complex for machines, such as phase separations, extractions, crystallizations, etc.

Additionally, self-optimization has shown promise in the automated optimization of single reactions, but there is a wealth of reaction data available that current algorithms are unable to utilize. Very recent work has used transfer learning techniques to accelerate optimization by leveraging data from similar reaction optimization campaigns, but this has mainly been demonstrated in silico,^189,190^ with one active learning example from Lapkin and co-workers with the focus of pH adjustment.^191^ There is also a significant opportunity for benchmarking these algorithms on both in silico and real-life experimental case studies to see how well they generalize to all classes of reactions.

## 6 Data-Driven Optimization

### 6.1 High-Throughput Experimentation

High-throughput experimentation (HTE) involves running multiple reactions in parallel, which is a useful technique for quickly exploring chemical space in a systematic and standardized manner.^192−200^ Traditionally, this process has been employed in the pharmaceutical industry for the parallel synthesis/assessment of chemical compounds on a variety of scales. This includes small, focused arrays of compounds for exploring structure–activity relationships (SAR) around a hit compound of interest, and the synthesis of thousands of compounds to populate high-throughput screening (HTS) libraries. In recent academic settings, HTE has also shown utility in the discovery of new chemical reactions, as this technique is well-suited for the discovery of unexpected reagent combinations enabled by the large number of reactions that can be run in tandem.^201−205^ For the purposes of this review, this section will focus on the application of HTE to accelerate the optimization of synthetic organic reactions. Herein, methods employed for the simultaneous exploration of chemical space using multifactorial optimization will be discussed, touching on the strengths and weaknesses of the different methods currently available to HTE practitioners.

#### 6.1.1 Multifactorial Optimization

**Figure 14:**
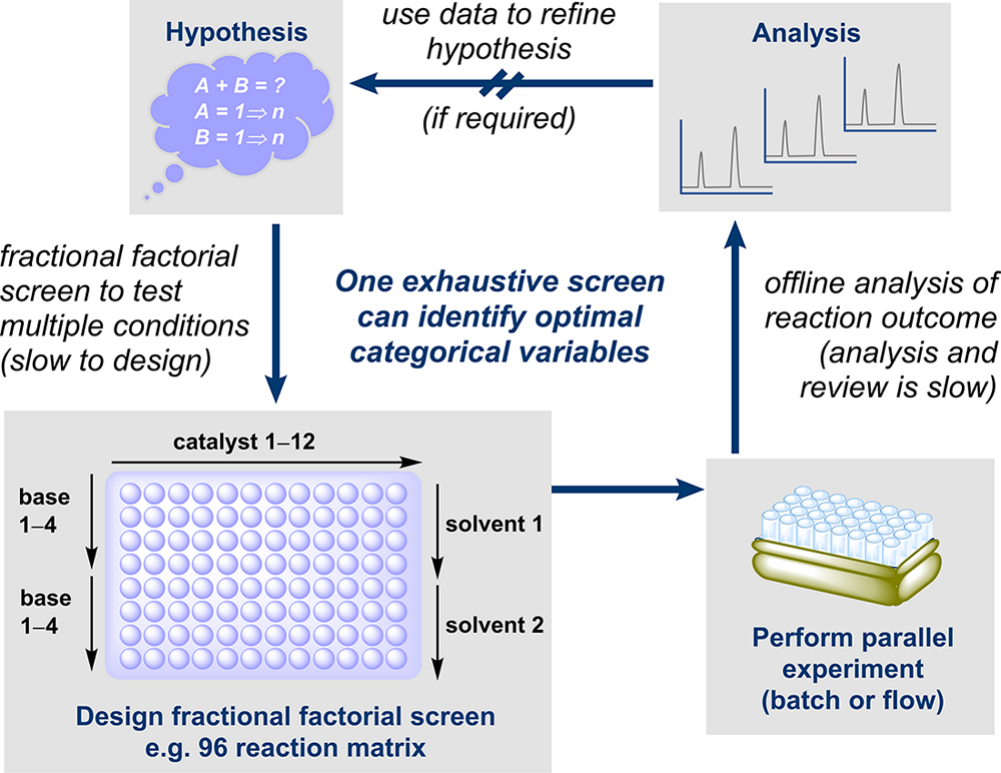
Schematic of a typical HTE workflow where a particular chemical process is optimized with respect to 12 catalysts, 4 bases, and 2 solvents.

With HTE, a large proportion of chemical optimization space can be examined at once in an “all vs all” manner, where one exhaustive (full factorial) screen may help to identify optimal conditions much faster and more efficiently than performing reactions individually (e.g., using OFAT). However, reaction optimization using a HTE methodology requires time-intensive reaction design from the outset, as several categorical variables (e.g., catalyst, solvent, base, etc.) may be varied at once.^192^ Commonly a fractional factorial approach is employed where a subset of variables are screened in a matrix array with all categorical variables compared against each other. Although this approach can be time-consuming to design and analyze, it is generally more cost-efficient than OFAT (see section 2) or other iterative optimization methods due to the miniaturized scale requiring less reaction material. An example HTE workflow is shown in Figure 14, where a chemical process is explored with each possible variation of 12 catalysts, 4 bases, and 2 solvents. HTE is a powerful resource that has seen widespread use in the pharmaceutical industry. It is employed in different areas of chemical development throughout the drug discovery process, from early stage preclinical development up to process optimization for clinical development, in-human testing, and subsequent market release.^193^

#### 6.1.2 Reaction Miniaturization

During the lead optimization phase of a drug discovery campaign, elaborated compounds (leads) produced in the development of a clinical candidate can be very time and resource intensive to make. High-throughput optimization techniques can be useful to optimize potentially intractable reactions such as transition-metal catalyzed cross-couplings of highly functionalized drug-like compounds, however, these elaborate pharmaceutical intermediates often cost more gram-for-gram than precious transition-metal catalysts that may be employed in their synthesis. By downscaling chemical reactions into a miniaturized format, one can increase the number of data points gained during a HTE screen without the associated impact on material cost.

For decades, biological assays have been routinely performed in plate-based formats on micro- or nanoliter scales in a parallelized high-throughput manner.^206^ This miniaturization approach not only allows for a greater number of experiments to be run in a material sparing fashion, but is also highly appropriate for automation. Hence, a number of robotic platforms have been developed by vendors (e.g., Tecan, Hamilton, Beckman Coulter Echo, SPTLabtech Mosquito) to facilitate the execution of multiple experiments at once in a Society for Biomolecular Screening (SBS) footprint microtiter plate (MTP). This data capture workflow was further streamlined with the advent of analytical systems equipped with autosamplers that can sample directly from the same footprint MTPs. The standardization of these plates to a universal footprint ensures interchangeability between different robotic systems, and this has undoubtedly had an enormous impact on the rate with which experiments can be performed and analyzed within the biological sciences.

Organic chemistry has been slow to adopt MTPs as, unlike biological experiments which are run under aqueous conditions at physiological temperature (37 °C), synthetic organic chemistry experiments employ a wide range of temperatures (cryogenic to elevated, e.g., −78 to 150 °C) and differing polarity solvents, some of which are incompatible with plastic MTPs. Accordingly, when chemists perform reactions in parallel, they tend to miniaturize their chemistry from round-bottomed flasks (RBFs) to 96-well plates and seldom downscale further to 1536-well MTPs. Table 1 explores some of the chemical limitations which need to be taken into consideration when miniaturizing chemical reactions into these plate formats. Table 2 explores how translating chemistry from common synthetic apparatus such as round-bottomed flasks into plate-based formats means that chemical synthesis can be economized, thereby increasing the density of information obtained from the same amount of material.

**Table 1:**
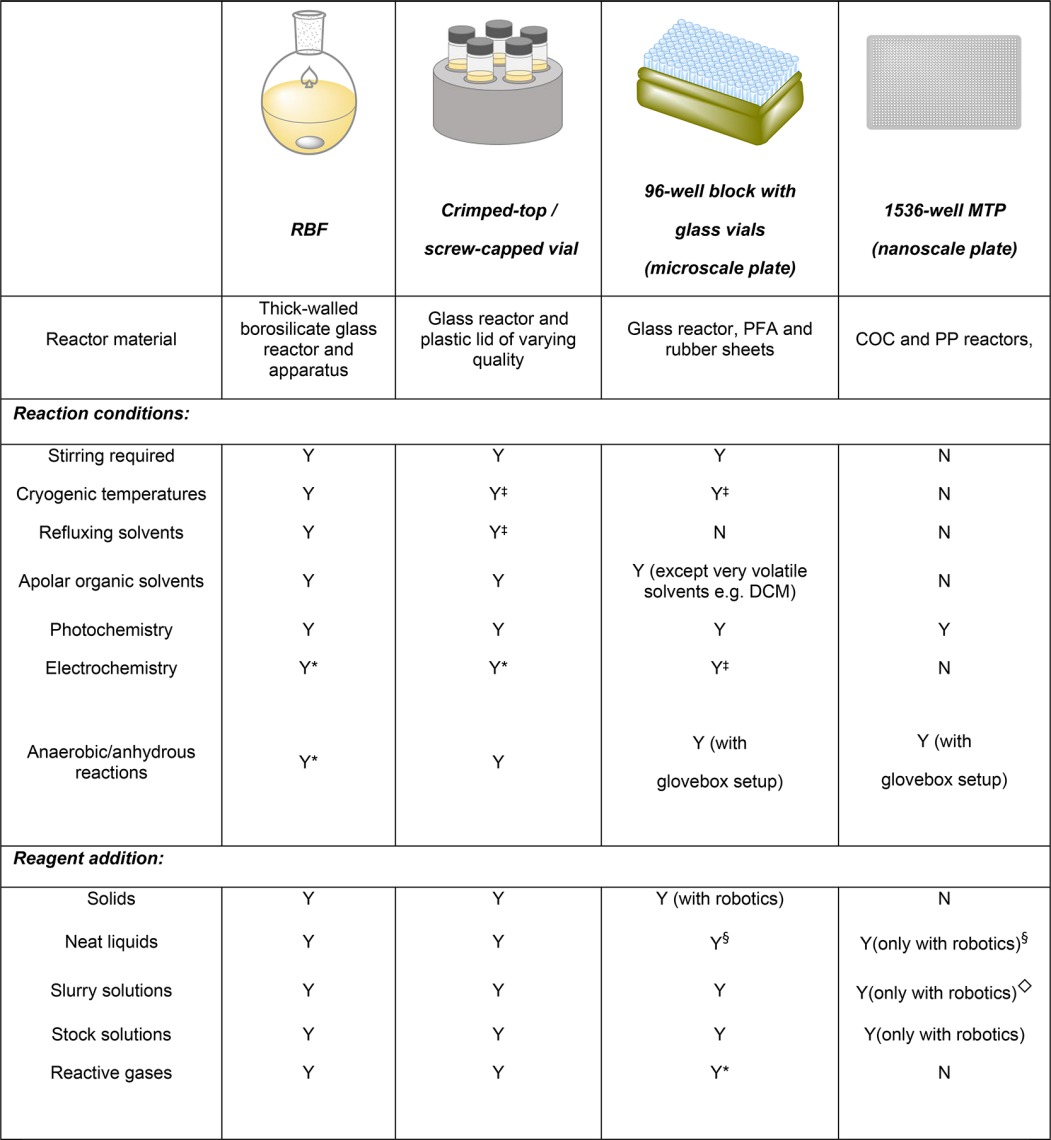
Chemical Reaction Limitations with Relation to Current Miniaturization Technologies^a^

a RBF, round-bottomed flask; MTP, microtiter plate; PFA, perfluoroalkoxy alkane; COC, cyclic olefin copolymer; PP, polypropylene; DCM, dichloromethane. *With specialized glassware or additional apparatus attached. ‡Vessel or equipment dependent. §Depending on amount required. ◇Reagent dependent.

**Table 2:**
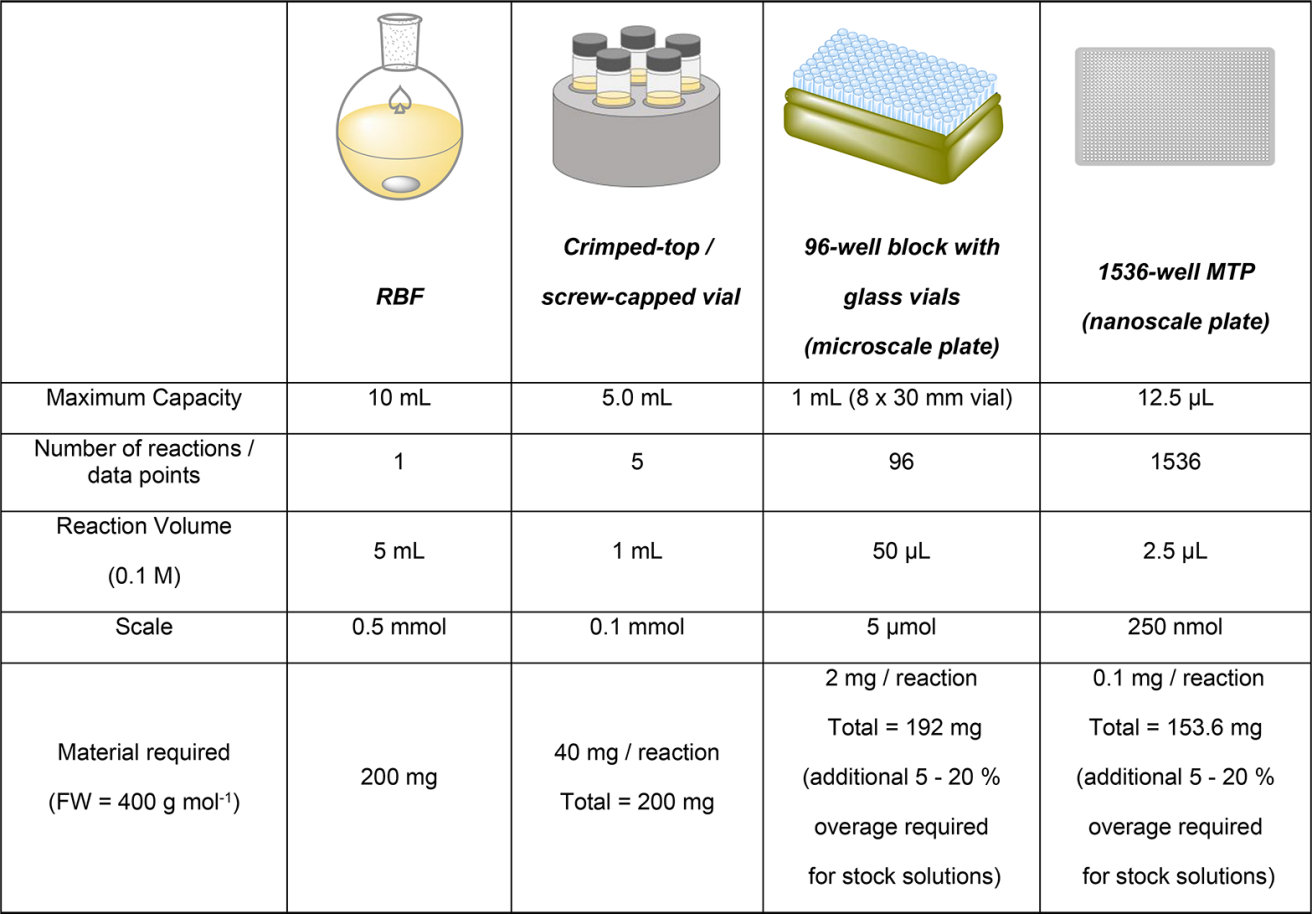
Physical and Pragmatic Reaction Constraints Using Miniaturization Technologies^a^

a RBF: round-bottomed flask; MTP: microtiter plate.

#### 6.1.3 Platforms for HTE Optimization

Platforms used for automated HTE can be broadly characterized into the following three formats: (1) plate-based reactors where individual reactions are either performed directly in the plate wells or within a glass vial insert, (2) flow-based platforms employing continuous^31,207^ or radial^208^ flow techniques, or (3) microfluidic (and droplet) reactors.^204^ These platforms have been utilized for a number of different applications including miniaturized synthesis and reaction discovery, but the examples shown herein are those which have been applied to reaction optimization only.^201−203,209^

##### 6.1.3.1 Microscale Plate-Based Optimization

Microscale plate-based chemistry involving 96 individual glass vials housed within a metal block (Tables 1 and 2) is one of the most routinely used methods for HTE reaction optimization. The apparatus required is relatively low cost, and liquids can be rapidly dosed using multichannel displacement pipettes and solid reagents (either as pure material or as ChemBeads)^210−213^ can be weighed out manually with small spatulas or using 3D-printed scoops,^196,210^ which can somewhat streamline the meticulous and demanding process. Unlike other nanoscale approaches in 1536 MTP, these 96-well reactors now allow an experimentalist to operate on a scale which is compatible with solid handling of chemical substances. Although the addition of solids to a microscale reactor can be more time-consuming than the addition of liquids or stock solutions, the ability to use solids is important as it broadens the variety of chemical reactions that can be performed, as not all chemical reagents can be effectively dispensed as stock solutions due to issues such as heterogeneity and chemical instability.

Another key benefit of working on microscale in 96-well reaction blocks is that the reactors themselves are suitable for a wider range of reactions than can be performed in plastic MTP reactors, the glass vial inserts in the 96-well plates exhibit good chemical tolerance akin to the traditional round bottomed flask, furthermore, they can be heated or irradiated, and the contents of the vials shaken on an orbital shaker or magnetically stirred with the addition or small magnetic fleas. Although automation is not necessary for reaction implementation using this setup (unlike the 1536-well MTP approach), the SBS footprint of these reactors means that they are compatible with a variety of different platforms for automated liquid and solid handling^214,215^ to streamline reaction implementation and optimization. The flexibility and accessibility of this 96-well approach has led to widespread usage with multiple literature examples for HTE optimization^216,217^ of a variety of reaction classes with selected examples shown in Table 3.

**Table 3:**
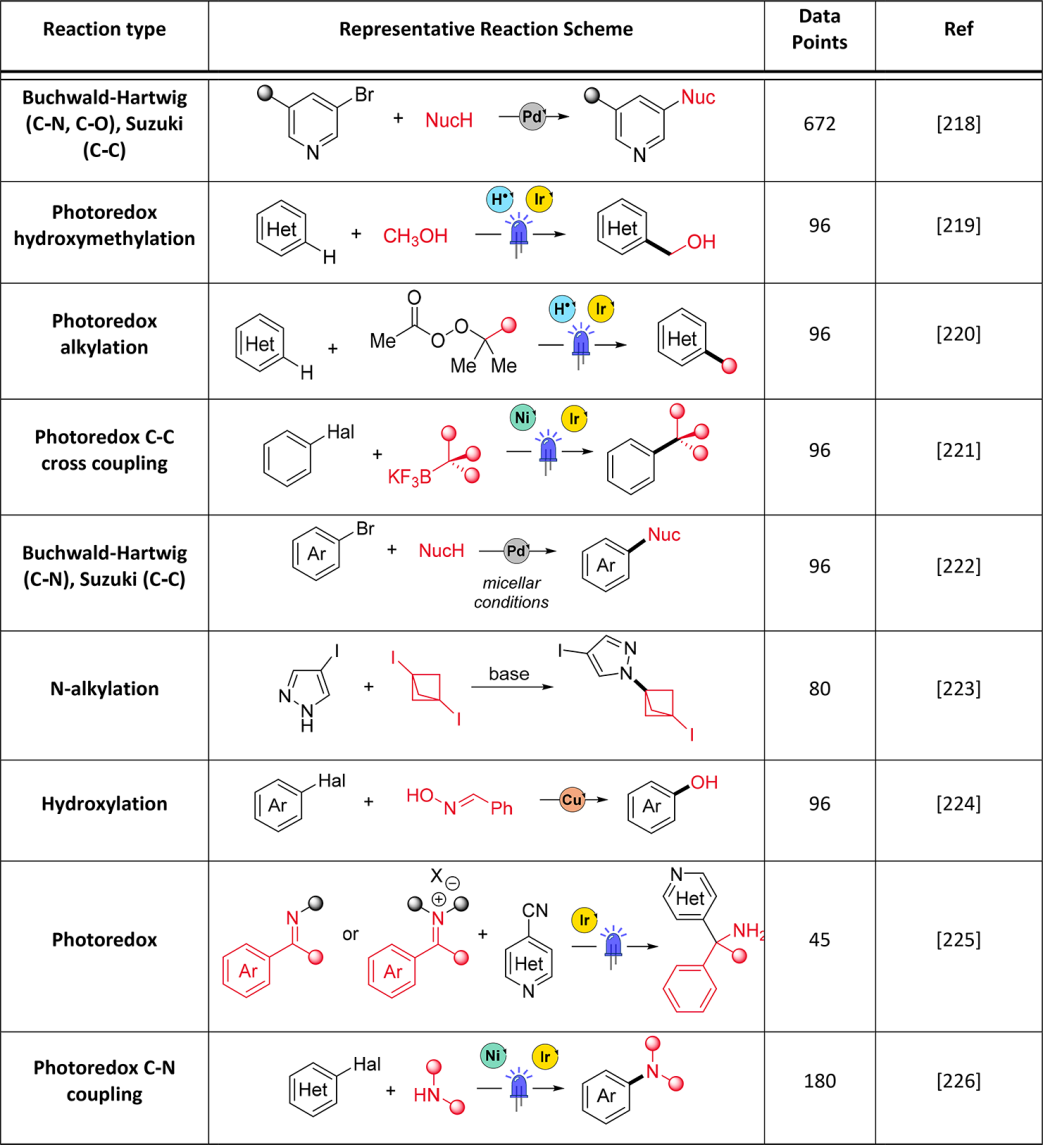
Examples of Different Reaction Classes Optimized Using the 96-Well Glass Inert/Metal Reaction Block Approach^218−226^

##### 6.1.3.2 Nanoscale Plate-Based Optimization

Nanoscale 1536-well MTP chemistry, recently referred to as ultraHTE,^198^ is a commonly reported HTE method which can facilitate the collection of thousands of data points within a multiparameter optimization.^227−229^ Due to the minute volumes of material employed in this approach, access to specialized liquid handling robotics is required. Examples include SPT Labtech’s Mosquito positive displacement multichannel pipetting system, which can dispense and aspirate volumes in the range of 25 nL to 1.2 μL, or Beckman’s Echo liquid handler, which can dispense volumes in the range of 2.5 nL to 5 μL using Echo Acoustic Technology. These platforms are increasingly used for nanoscale (or even picoscale) synthesis of pharmaceutically relevant compounds with examples of also incorporating nanoscale biological screening in a “direct-to-biology” approach.^230−239^

The Mosquito/1536-well MTP platform pioneered by Merck Research Laboratories is prevalently reported in the literature for nanoscale optimization. Although this approach has limitations related to the breadth of chemical reaction types that can be performed in 1536-well MTPs (Table 1), ultraHTE has been successfully employed on multiple occasions for the multiparameter optimization of a variety of different reaction types from the medicinal chemistry toolbox.^17,240−242^ These reactions include Suzuki cross-couplings, reductive aminations, *N*-alkylations, nucleophilic aromatic substitutions (S_N_Ar), etc., as well as transition-metal catalyzed couplings on pharmaceutically relevant molecules such as the Pd-catalyzed Buchwald–Hartwig amination, metallophotoredox C–N and C–O bond formations, and C–H functionalizations to furnish C(sp^2^)–C(sp^3^) bonds (Table 4).

**Table 4:**
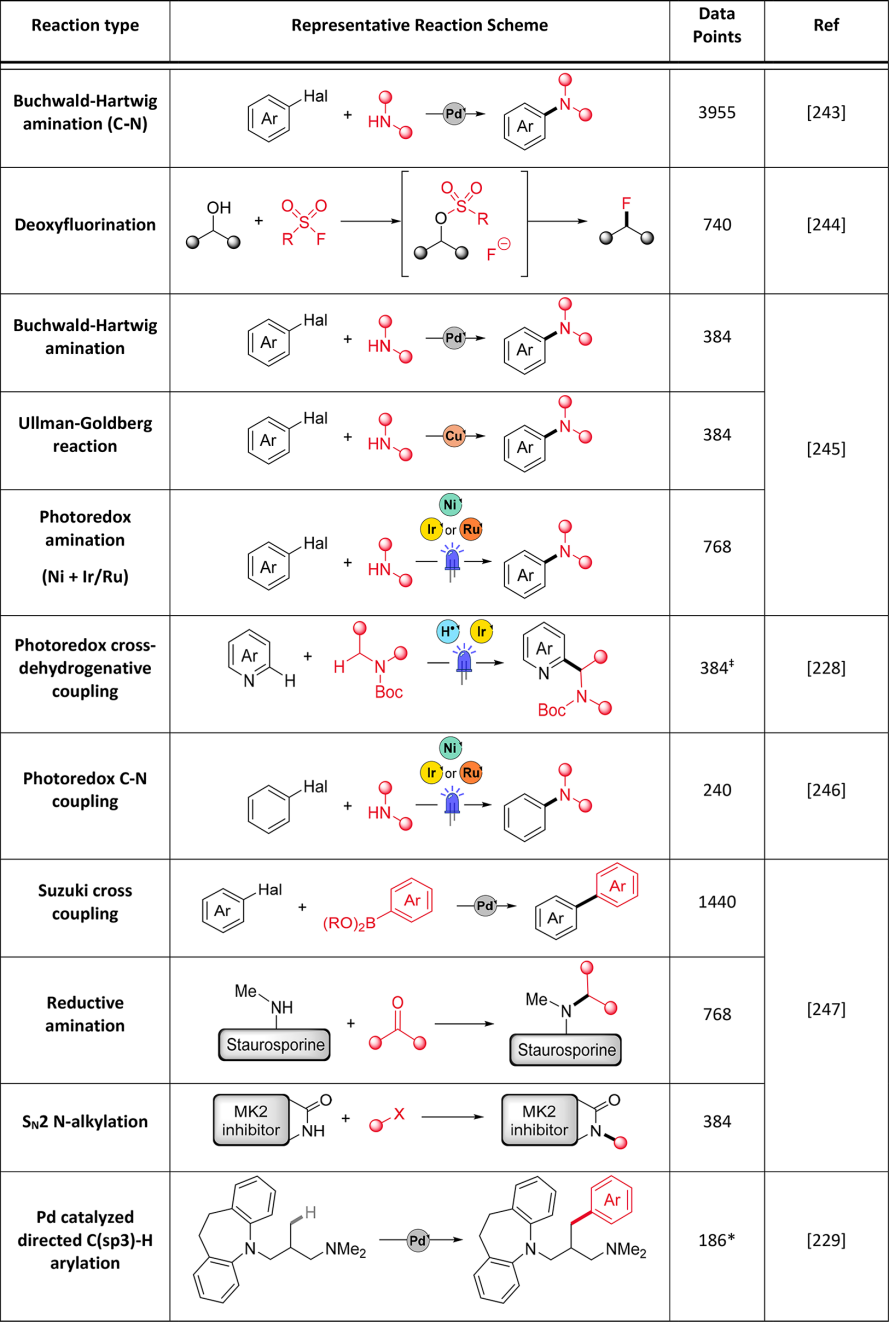
Examples of Different Reaction Classes Optimized Using the Mosquito/1536 MTP UltraHTE Platform^a^^228,229,243−247^

a ‡, in duplicate; *, in quadruplicate.

##### 6.1.3.3 HTE Plate Analysis

**Figure 15:**
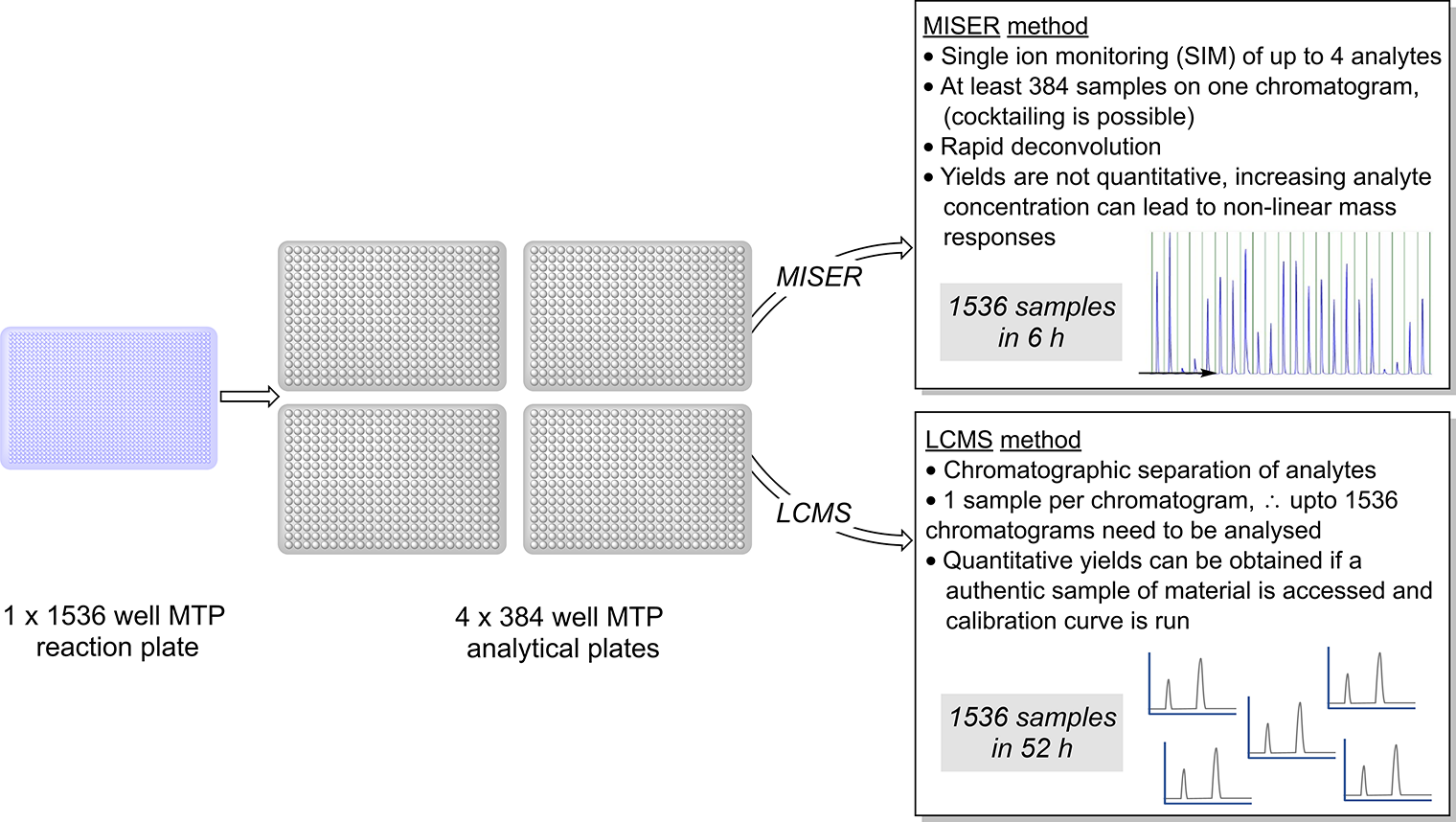
Routine HPLC-MS kit equipped with a single-quadruple MS can be used for analysis of ultraHTE reactions direct from 384-well MTP. Two techniques can be employed, either the multiple injections in a single experimental run (MISER) method or a more traditional LCMS/UV method, however, there is a trade-off between speed and the level of quantification that can be achieved.

When using HTE to optimize chemical reactions, it is important to not only consider the practicalities of how to perform multiple reactions in tandem, but also how to analyze and deconvolute the reaction outcome in a similarly high-throughput manner. Without careful consideration of the overall experimental and analytical workflow, a bottleneck can occur.^248,249^ In the past decade, several ground-breaking developments in the analytical sciences have occurred which now permit the ultrafast analysis of high-throughput reaction screening at increasingly impressive speeds. For example, techniques like matrix-assisted laser desorption/ionization (MALDI),^243,250,251^ desorption electrospray ionization (DESI),^252−254^ and acoustic ejection MS (AE-MS)^233,255−257^ have been reported for the high-throughput data acquisition of 1536 reactions in under 10 min and requiring only nanolitre volumes of crude reaction mixtures. These techniques are rapid, and in some cases the analysis can be performed directly from a 1536-well MTP, however, the equipment required can be expensive. Other options for rapid analysis which can be performed on standard UHPLC hardware interfaced with a single-quadrupole mass spectrometer is the Multiple Injections in a Single Experimental Run (MISER) technique developed by Merck.^227,258^ This flow-injection analytical method injects multiple samples back-to-back with limited or no chromatographic separation and uses single-ion monitoring (SIM) to detect analytes. With inexpensive UHPLC-MS equipment, run times are reported to be as low as 10 s per sample, resulting in the data acquisition of 1536 samples possible in around six hours. The main drawback of this technique compared to the ultrafast MS methods is that reformatting from a 1536-well MTP to four 384-well MTPs (Figure 15) is required as currently no commercial LC-MS autosamplers can accommodate these plates. Furthermore, when compared to methods like AE-MS, ion suppression from common diluents (like DMSO) can be problematic.^248^

Although the development of these rapid analytical techniques has had a notable impact on the speed at which analytical data from HTE reactions can be acquired, there remains a trade-off between (1) speed of acquisition, (2) sensitivity of the technique employed, (3) the amount of structural information gained from the technique, and (4) the level of quantification that can be achieved. With ultrafast techniques like MALDI, DESI, AE-MS, etc., quantitative data can be acquired providing an internal standard is added to the analytical samples prior to analysis, an authentic sample of the analytes can be obtained, and a calibration curve run to compare measured analyte response and concentration to henceforth determine yield. This calibration is necessary as ionization responses of different analytes can be nonlinear and difficult to compare reliably. However, even if authentic samples of the analytes can be sought, other phenomena like ion suppression can add additional complications to the level of accuracy of yield assessment that can be realistically achieved with MS-based techniques. More conventional LC-MS methods which chromatographically separate analytes and use UV absorbance (e.g., at 210 nm) to determine analyte concentration tend not to suffer from the same issues as MS-based techniques (e.g., nonlinear responses at increased concentration and ion suppression). However, these methods require longer run times (cf MISER) and may require optimization of the elution gradients to ensure peak separation. Thus, although this technique can afford increased confidence in the level of quantitation that can be obtained, this comes at the cost of prolonged analysis times for both acquisition of data and deconvolution of reaction outcome (Figure 15).

##### 6.1.3.4 HTE and Continuous Flow

Flow chemistry is a technique that is highly applicable for optimization methods such as self-optimization, as discussed in section 5. Commercially available equipment such as the Vaportec R-series or systems from ThalesNano or Uniqsis, combine an autosampler for reagent selection and following injection into the flow reactor, in-line analytical equipment can also be coupled for in-line analysis. These setups are well-suited to automated synthesis, however, there are currently no off-the-shelf continuous flow setups which can rival the throughput of plate based HTE, and consequently there is a scarcity of reports of HTE used in continuous flow. More often continuous flow is used for the scaling up or continuous variable optimization of HTE hits identified in plates,^228,259,260^ however, before this can be accomplished, reoptimization is often required as chemistry seldom translates directly from batch or plate to flow.^228,230^

**Figure 16:**
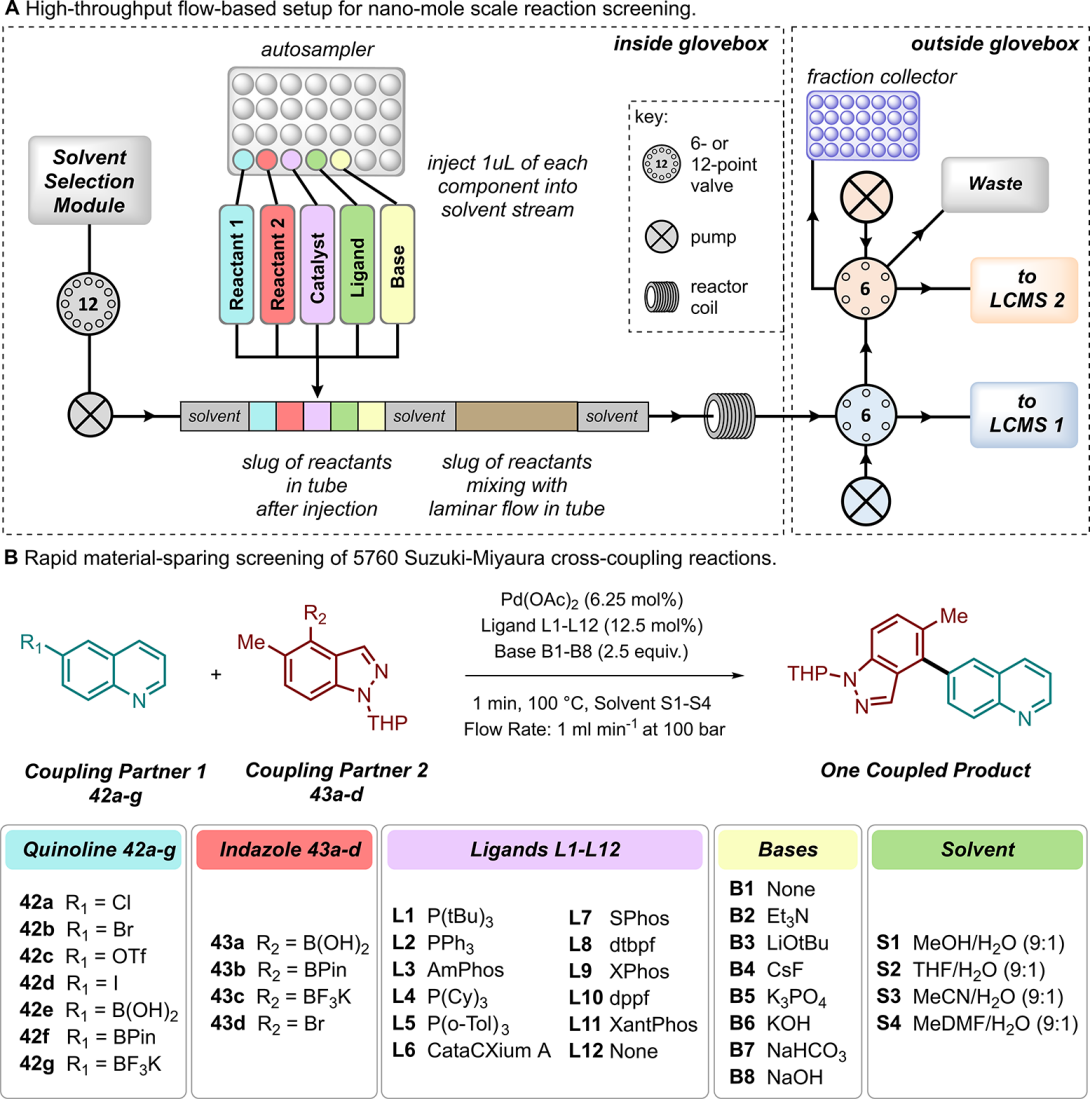
Platform for automated nanomole-scale reaction screening and micromole-scale synthesis in flow developed by Pfizer and reported for the optimization of a Suzuki reaction.^207^

Pfizer has recently reported a bespoke flow platform for nanoscale HTE based on modified HPLC equipment, as shown in Figure 16.^207^ The autosampler which would normally house analytical samples is repurposed to hold up to 192 source vials containing stock solutions of reagents, which is a significant increase in capacity compared to existing commercial platforms. The reaction segment is assembled in ∼45 s, where 1 μL of each reagent is aspirated and then the whole slug is injected into a flowing solvent stream and delivered into the reactor with approximately one-minute residence time. The reaction mixture is then directed into a 96-well plate fraction collector and subsequently analyzed via LCMS. This system has the benefit of not only containing several switching valves which can permit selection of a several solvents but can also direct the reaction slug to one of two LCMS machines for analysis. This platform enabled the execution and analysis of around 1500 reactions in a 24 h period and was also subsequently employed for the optimization of a photoredox catalyzed decarboxylative minisci-type C–H arylation to afford bicyclo[1.1.1]pentane containing compounds, which are medicinally relevant and traditionally challenging to synthesize.^261^

A workflow recently reported by Bourne and co-workers also shows the use of a bespoke flow reactor platform for library synthesis and optimization.^262^ This work utilizes “stopped-flow” experiments and machine learning models to map chemical reactivity and synthesize diversity-oriented libraries. This led to a system that can predict optimal synthesis conditions with 92% accuracy and a dramatic increase in the success rate of initial library screens for reactivity while achieving a 90% reduction in reagent consumption when compared with continuous flow.

#### 6.1.4 HTE for Data Set Generation

Data driven optimization involves the use and analysis of relevant data to guide decision making in pursuit of the global optima for a given process. In practice, the success of this endeavor is highly dependent on the quality of data utilized, a factor that is increasingly important for autonomous reaction optimization systems using algorithmic, generative, and machine-learning (ML) derived models. One of the biggest challenges currently faced in this area is the acquisition of abundant and high-quality data sets. The availability of reaction data accessible to cheminformaticians has dramatically increased as online databases such as SciFinder-CAS, Reaxys, and the United States Patent and Trademarks Office (USPTO) have become accessible, as discussed in section 6.2. However, there are limitations to the condition of data obtained from these databases, as they are collated from numerous sources and usually extracted using text mining software which can lead to poorly standardized data and noisy data sets. Automated HTE offers the potential to acquire high-quality data sets which are well-standardized and most importantly, include negative data points, however, there is currently a scarcity of these data sets which are publicly available.

Although data sets compiled from mining patent repositories and published reaction literature are extensive and cover a wide-ranging number of reaction classes, there are limitations to the quality of data as it is merged from many different laboratories using different methods and different equipment for quantification. Deviations in reaction outcomes of the same reaction might arise from human error in the laboratory, transcription, or limitations of the text mining software, varying analytical methods and different reaction scales (mg to kg scale). Moreover, it must be noted that generally literature data is biased toward higher yields as low yielding outcomes are often not reported. In detail, NMR yields are usually calibrated to an internal standard within the reaction sample or probe, although isolated yields are considered the “gold standard,” these can be limited by sample recovery success which is highly dependent on the purification techniques used. UV- (e.g., HPLC-MS diode array responses) or mass-based techniques such as HPLC-MS or GC-MS can also give varying degrees of quantification as UV of MS responses can vary between compounds and purely quantitative results are only achieved by comparing the mass or UV response to an analytical calibration curve. By contrast, HTE offers an opportunity to generate data with a high degree of standardization, reactions tend to be performed and analyzed on the same scale with the same equipment using the same stock solutions. The power of this approach is apparent when comparing the variance in yield prediction models trained on HTE-derived data for specific reactions compared to a much larger data set from USPTO as highlighted by Reymond and co-workers.^263^

#### 6.1.5 HTE Outlook

The past decade has seen a shift in the application of HTE from the generation of chemical samples for HTS libraries to conditions screening and optimization of chemical reactions by taking advantage of the ability of HTE to rapidly survey a wide area of chemical space using a small amount of material. Recent advances in HTE and the translation of nanoliter robotics like the SPT Mosquito and Beckman Echo, originally designed for biological assays and now being applied to parallelize organic synthesis, has fueled a rise in reports of ultraHTE where hundreds or thousands of chemical reactions can be executed in parallel on a miniaturized scale. Although limitations still exist with regards to the types of chemistry that are amenable to these plate-based formats, further development to increase the flexibility and generality of chemistry which can be performed in this fashion will be a benefit to the field of reaction optimization by HTE. Furthermore, as HTE plates are sealed and heated as a block, flexibility in reaction times, reagent equivalents, and temperature controls are limited as the entire plate is subjected to the same conditions. This means that although promising categorical variables can be identified as “hits” for promising potential optimal conditions in reaction optimization, many continuous variables must then be optimized upon scale-up to more traditional batch or flow laboratory scales.

As discussed herein, another advantage of translating organic chemistry to a miniaturized HTE configuration is the ability to rapidly generate large amounts of standardized data in a relatively short time frame creating information density for a desired reaction or class of substrates. Currently, there are few examples of utilizing HTE-generated data to train ML models and the increased availability of more publicly available data sets would be particularly useful to the machine learning in the chemistry community. In this regard, challenges exist around the extent of quantitative data which can be obtained using an ultraHTE approach; currently there is a trade-off between speed and quantitation, and there are also constraints around the number of different analytes that can be analyzed in a quantitative manner in tandem. Recently, there has been significant progress in the field of analytical chemistry and robotics, which have directly facilitated the renaissance of HTE in the field of organic synthesis and further developments are sure to modernize the area further and increase the number of data sets available to data scientists.

### 6.2 Data Mining, Machine Learning, and Optimization Benchmarking

Machine learning (ML) has already revolutionized various areas, such as image recognition,^264^ natural language processing,^265^ and autonomous driving.^266^ Within the field of organic chemistry, ML also represents an emerging tool, particularly for prediction tasks such as retrosynthesis, optimal reaction conditions, or reaction outcomes. It is then also possible to use these predictions from ML to influence starting points and process bounds for real-world optimizations, whether in self-optimization, HTE, or otherwise.

**Figure 17:**
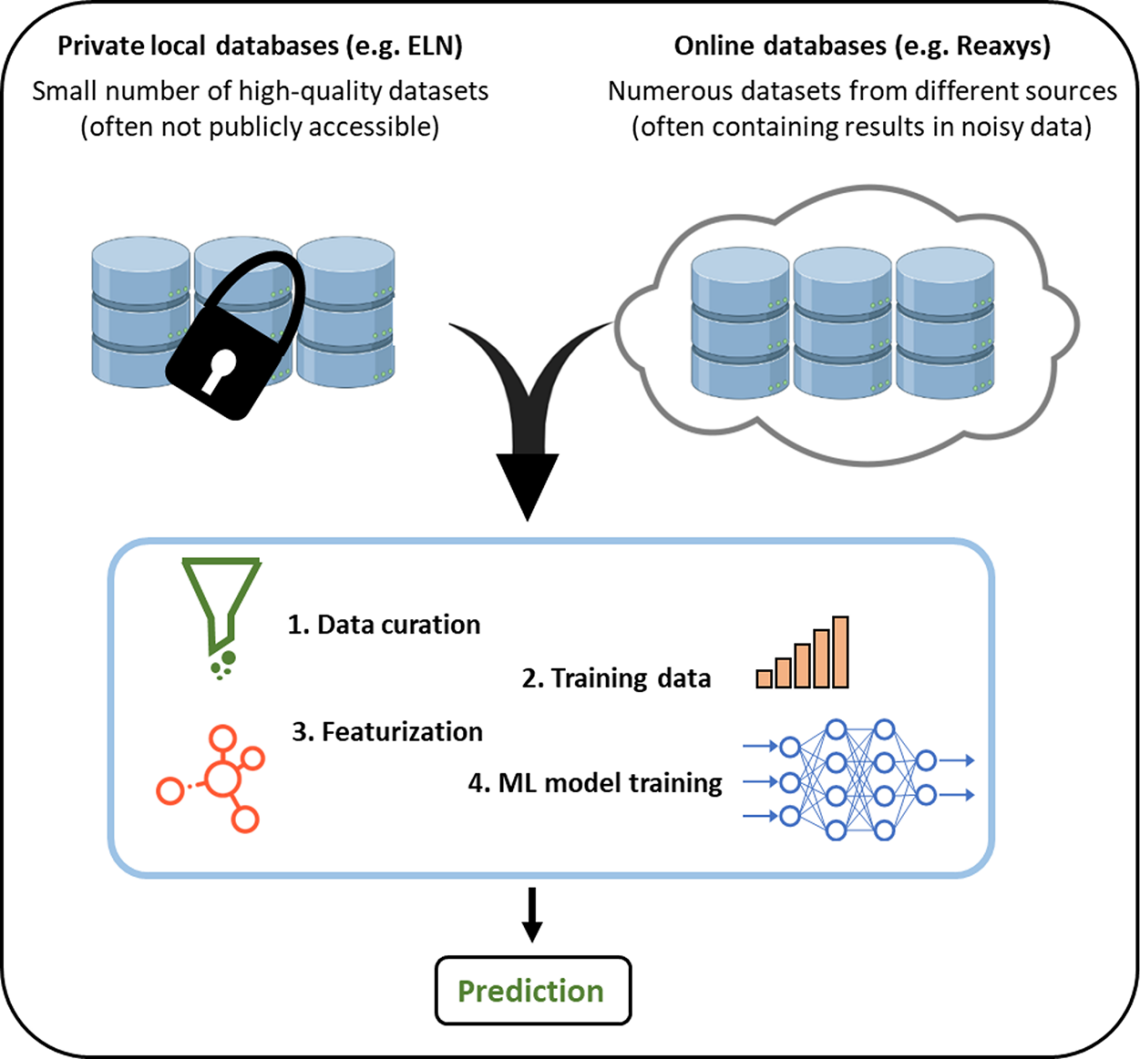
Schematic of the data collection and model training steps of using machine learning for reaction optimization from public or private reaction databases.

The prediction of reaction outcomes at specific reaction conditions or direct prediction of reaction conditions are relevant and particularly attractive for reaction optimization. As shown in Figure 17, this problem is subdivided into data collection and model training. Once a data set is extracted from a high-throughput experiment or a reaction database, a chosen ML model is trained to predict reaction conditions or outcomes. Typical inputs for ML models include continuous reaction parameters such as reaction time, temperature, or reagent equivalents and categorical parameters involving the choice of catalyst, reactants, or bases. In the case of reaction outcome prediction, outputs are typically the targets of reaction optimization such as yield, conversion, or enantiomeric excess (ee). The predictive performance of these trained models is subsequently evaluated on unseen test data. Thus, one main goal is to develop modeling strategies that capture the correlation between reactants, reagents, and chemical reactivity to avoid brute-force laboratory screening which can be wasteful (particularly without the use of HTE equipment). Choosing the best molecular parametrization is a key aspect of achieving that goal.

#### 6.2.1 Molecular Parameterization

Molecules must be translated to a machine-readable, typically numerical, format that can be used as an input for ML models, prior to their use. We refer to this translation process as molecular parametrization as it aims to capture relevant molecular properties for a particular reaction. For different chemical transformations, different properties can influence reaction outcomes such as steric hindrance of a functional group or electronegativity of neighboring atoms. Moreover, the parametrization strategy should also be chosen to allow optimal compatibility with the ML model used, as prediction performance will depend on the compatibility between input format and ML model.

The baseline parametrization method for representing chemical inputs is one-hot encoding (OHE). A one (1) or a zero (0) represent the presence or absence of specified reaction components respectively: no chemical information is encoded. This approach has been shown to be effective for a variety of chemical tasks, including yield prediction, but cannot extrapolate to new parts of chemical space.^229^

Extended-connectivity fingerprints (ECFP) is a parametrization method that captures atom types, neighboring connectivity relationships, bond types, and represents the outcome in a machine-readable one-dimensional bit-vector. Circular fingerprints (e.g., Morgan fingerprints) are generated by (1) assigning identifiers to each atom in the molecule, (2) updating each atom’s identifiers depending on the neighboring atoms, (3) removing duplicates, and (4) compressing the data into a vector of set length, e.g., 1024 bit (a number of zeros and ones).^267^ One of the advantages of these fingerprint based methods is that they are considered cheap features for modeling; their generation does not require a vast amount of computing power/time. Yet, their ability to explicitly capture molecular properties (e.g., sterics, electronics) of molecules is limited. Typically, models that use fingerprints develop knowledge in an indirect manner, such as an implicit understanding of electronegativity associated with different halides, for example.^268^

A much more comprehensive parametrization approach is calculating molecular descriptors using density functional theory (DFT). DFT can be used to determine the ground/excited state of molecules and thus offer fundamental insights into geometric and electronic properties.^229^ As a result, DFT can be used to calculate descriptors that quantify the specific chemical properties of the given set of ligands such as the bulkiness of a molecule or electronegativity of atoms within a molecule.^269^ However, DFT calculations for large libraries are often more time-consuming than actually running the corresponding reactions in a high-throughput screening format.

More recently, parametrization work has utilized neural networks to achieve the tailored nature of DFT descriptors without the computational expense. This work is divided into two approaches: natural language processing models and graph neural networks. The former leverages recent advances in language models such as transformers,^270^ where results can be achieved by training a model to predict the next word in a sentence across a wide variety of texts. Because chemistry can be represented as a language in the form of simplified molecular-input line-entry system (SMILES),^271^ a language model can be trained to predict the next atom in a molecule when given only a portion of the molecule, thereby saving computational expense.^272^ Because the model must understand a significant volume of chemistry to be able to predict a SMILES string, its numerical output can be used as a “learned fingerprint” for other prediction tasks.^273^ Furthermore, the learned fingerprint can be tuned for each downstream task such as yield prediction using standard neural network training.

Alternatively, graph neural networks represent a molecule as an interconnected network of atoms and bonds. These networks can be trained to produce a “learned fingerprint” for prediction tasks. One of the most widely used forms of graph neural networks in chemistry are message passing neural networks (MPNNs), which learn relationships between neighboring atoms through iterative “messages” passed along bonds.^274,275^ MPNNs have been extended to generate fingerprints for reactions, with state of the art results.^276^ An overview of these techniques is shown in Table 5.

**Table 5:** Overview of the Commonly Used Molecular Parameterization Techniques for Modelling Chemical Data

parameterization method	information captured	data type	example data
OHE	existence/absence of a component	binary encoding	[0 0 0 1 0 0 0]
molecular fingerprints	atom type, atom count, chemical structure, connectivity	binary encoding	[1 0 0 1 1 0 1 0 0... 0 1]
DFT descriptors	interatomic information: length, angles, volumes	numerical values	0.001342, 45, ...
Electronic Information: Charge Distribution			
learned representations	connectivity and potentially atom and bond	numerical values	0.001342, 45, ...

#### 6.2.2 Prediction of Chemical Reaction Yields from High-Throughput Experiments

Reaction outcome prediction has primarily been carried out on data obtained via HTE or similar techniques for generating consistent data sets. Doyle and co-workers trained a random forest (RF) algorithm on HTE data of a Buchwald–Hartwig reaction, aiming to generate an automatic feature generation algorithm. They demonstrated success using their approach, which was trained on 5% of data and outperformed linear regression trained on 70% of data.^243^ Subsequently, Hirst and co-workers continued the work by Ahneman by using another machine learning technique, support vector machines (SVM), in which they demonstrated improved prediction performance.^277^ Glorius and co-workers successfully boosted this prediction performance using a concatenation of fingerprints.^278^ Doyle and co-workers then used a combination of fingerprint based and DFT based descriptors for the prediction of reaction performance for a deoxyfluorination HTE data set, thereby guiding the search toward high yielding conditions.^279^ Overall, due to the consistency of HTE generated data sets, good results could be achieved with regard to the predictive performance of the applied ML models.

#### 6.2.3 Reaction Databases

**Figure 18:**
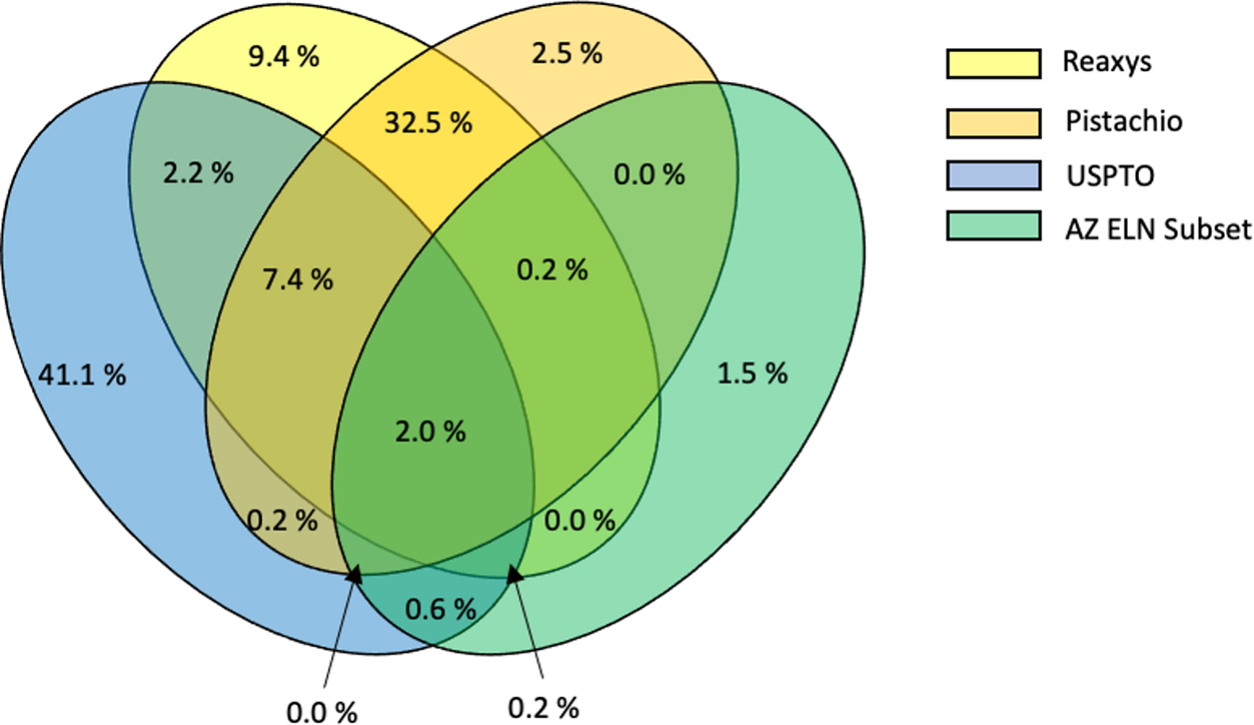
Illustration of the overlap of chemical reaction databases (Reaxys, Pistachio, USPTO, and a subset of AstraZeneca ELN13).^284^

While most of this covered literature used data solely generated via HTE or flow chemical platforms for training ML models to prediction chemical reactivity, an increasing effort is made in developing predictive models with chemical data mined from online databases. Reaxys, a commercial database by Elsevier, and the United States Patent and Trademark Office (USPTO) reaction database are the two most used sources for data-mined applications. The Reaxys database is proprietary and contains information on more than 56 million reactions from over 16 000 journals. In contrast, the USPTO database is accessible to the public and contains chemical reactivity information obtained from over nine million patent applications.^280,281^ Additionally, Pistachio represents a commercially available data set, based on USPTO data, electronic lab notebook (ELN) data, and information obtained from journals or other patent literature containing 13.3 million reactions.^282^ Most recently, the open reaction database was created to build a standard format and open-access location for reaction data, which represents a large shift in fair data accessibility.^283^Figure 18 illustrates the information overlap between several of these reaction databases.^284^

#### 6.2.4 Data Preprocessing

To build predictive models based on reaction databases, one of the most significant challenges is extracting data into a standard format amenable to machine learning. The database providers mentioned perform some amount of preprocessing to extract the data into a common format. However, the databases often do not capture important details of the procedure such as the reaction temperature, workup protocol, or analytical results. Furthermore, the same reagent can be represented in different ways across the database (e.g., metal catalysts can be represented with their ligands or as separate components). To overcome this issue with data quality, recent work has developed deep learning models that can extract text descriptions of synthetic procedures in a standard format.^285−287^

Subsequently, the data must then be filtered, in particular, as noted by Varnek and co-workers, there are many duplicate reactions in databases.^288^ This is often caused by scientists using the same procedure for a standard reaction as is reported in the literature. Therefore, a filtering process often includes removing duplicate reactions, discarding reactions with missing key reactants or reagents (e.g., a Suzuki reaction should always have an organohalide and boronic acid), and excluding reactions without a numerical yield. This filtering process can often result in less than the 30% of the original extracted data being utilized for machine learning. For example, Reymond and co-workers created a data set of Buchwald couplings based on data extracted from several databases and, after filtering, only 15% of the original reaction records remained.^289^

#### 6.2.5 Machine Learning for Reaction Condition Prediction

**Figure 19:**
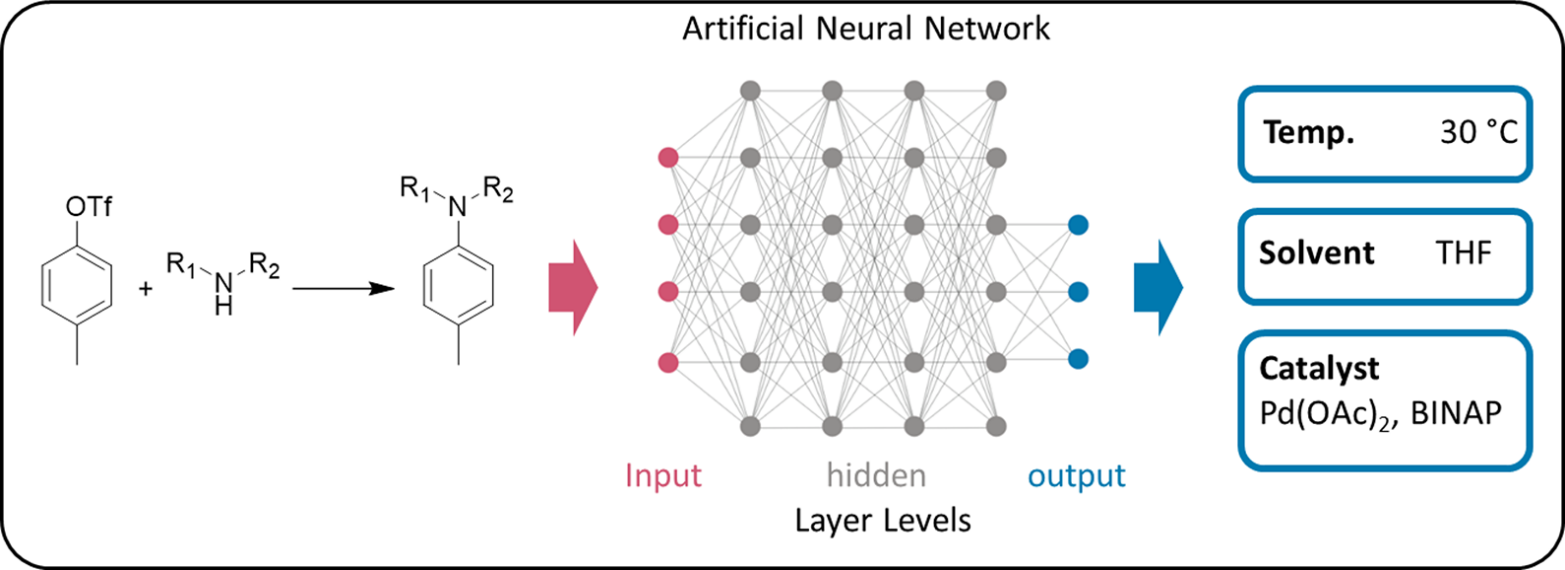
Demonstration of the architecture used by Jensen and co-workers for predicting reaction conditions.^290^

Upon data set extraction from the literature, machine learning models can then be used to predict reaction conditions directly given a set of reactants. The first examples of such a model were developed by Jensen and co-workers, who used a feed forward neural network to directly predict reaction conditions given the difference in the ECFP fingerprints of the products and reactants, as shown in Figure 19.^290^ Their neural network architecture was designed to reflect a chemist’s intuition. Often, catalysts are selected followed by solvents, reagents, and temperature, so Jensen and co-workers’s network first predicted catalysts and then conditioned each further prediction (solvents, reagents, and temperature) on the prior ones. On unseen reactions, the neural network’s top-3 predictions included those used in the literature with 50% accuracy. Furthermore, the correct catalyst was selected with over 93% accuracy.

Approaches that directly predict reaction conditions do not account for the yield of the reaction; instead, these techniques aim to predict the conditions used most often in the literature. The downside of this approach is that optimal reaction conditions for a particular transformation often require going beyond the standard conditions used for a transformation. Therefore, reaction condition prediction models might be used to give suggestions of starting points for further optimization rather than predicting optimal conditions directly.^291^

In addition to predicting reaction conditions, machine learning models can be trained to predict the likely products of a reaction given the reactants. A variety of models have been trained for this purpose, ranging from natural language processing models (e.g., transformers)^292^ to custom neural network architectures.^293^ Furthermore, machine learning models can be used for retrosynthesis, which has been reviewed extensively elsewhere.^294^

#### 6.2.6 Future Directions

Thus far, ML has been applied to yield prediction and reaction condition prediction, both of which have potential use for reaction optimization. Scientists can use yield prediction models to narrow the potential optimal sets of conditions for a reaction without brute-force screening. Recent work by Grzybowski, Burke, and co-workers showed this by highlighting the use of ML and closed-loop optimization to identify general high-performing reaction conditions for a Suzuki–Miyaura coupling.^295^ These ML models can therefore be combined with the optimization techniques described in section 5 to automate the identification of optimal reaction conditions with viable starting points.^15^

Currently, ML models have been very successful in reaction outcome prediction on HTE data sets for a single reactant pair. We foresee that leveraging a broad selection of data from targeted experiments could aid general prediction of key reaction outcomes for specific classes of reactions. Reymond and co-workers attempted to build a general yield prediction model based on the USPTO data set, but their model has low predictive quality due to the sparse nature of the USPTO yield data.^263^ Therefore, more high-quality data sets with reaction outcomes recorded and further model development are needed to create general ML models for reaction optimization. If successful, this research could be transformative in reaction optimization and transition the field to more direct predictions of optimal reaction conditions. For a recent review of in-depth modeling of HTE data sets, also refer to Jensen and co-workers.^296^

## 7 Scale-Up and Manufacture

Lab-scale reaction optimization studies focus on improvements in reaction yield and purity, optimization of reaction cost and greenness, and development of the optimal workup/separation approach, whereas subsequent process design steps (necessary for scale-up) need to address a different set of requirements.^297^ The ability to deliver commercially relevant quantities of product, with an acceptable investment, operational and environmental cost, as well as process safety, are the focus of this stage of process design. Furthermore, continuous fulfilment of quality critical parameters is investigated at this step, fundamental for pharmaceutical and fine chemicals industries.^298^ Development of processes meeting these criteria is defined as scale-up and involves determination of the critical scale-dependent factors that would affect the choices of the most functional reactor architecture, process conditions, and separations steps. These are the next steps from bench-scale reaction optimization and understanding how previously obtained optimal conditions may change is crucial to the successful scale-up of chemical processes.

Scale-up implies the attainment of significantly larger product throughputs compared to laboratory reaction discovery or reaction development studies. This is traditionally realized by deploying significantly larger reactors with very different gradients of temperature, pressure, and reactant concentrations to the small-scale processes.^299^ Therefore, it is often very difficult to reproduce at scale exactly the same conditions obtained when using small-scale equipment and the most promising operating conditions determined during lab-scale optimization studies do not necessarily yield an optimal large-scale process. As a result, many initially promising discoveries and reaction routes may not achieve broad deployment in industrial production.^300,301^ While large companies can invest vast resources into solving scale-up related challenges,^302^ smaller research organizations such as start-ups or academia might not be in place to allocate such resources, limiting the technology-readiness level of developed reactions.^297^

Determining the optimal reaction conditions for the scaled-up reaction involves careful consideration of how the processing conditions change in space and time and how these changes relate to the time scale of the molecular events driving the reaction. Elucidating these phenomena requires simultaneous insights into the kinetics, heat transfer, and mass transfer happening inside of the reactor. In contrast to exhaustive scale-up guidelines formulated for chemical engineers and process chemist experts,^303−305^ this section discusses scale-up on a conceptual level, providing insight for chemists working on molecular discovery and benchtop optimization. We anticipate that the consideration of scale-up challenges and complexity in the early stages of process optimization can help to guide the laboratory studies toward the achievement of metrics meaningful for large-scale plants and consequently accelerate the process of launching new molecules and products to market.

The following subsections briefly discuss scale-up considerations to be addressed during benchtop experiments, as well as which phenomena change during the transition to larger reactors and how to quantify these changes. Furthermore, we introduce two strategies used to develop large-volume processes: scale-up and numbering-up, as well as guide the user toward a choice of applicable equipment for each case.

### 7.1 Scale-Up Considerations within Reaction Optimization

**Figure 20:**
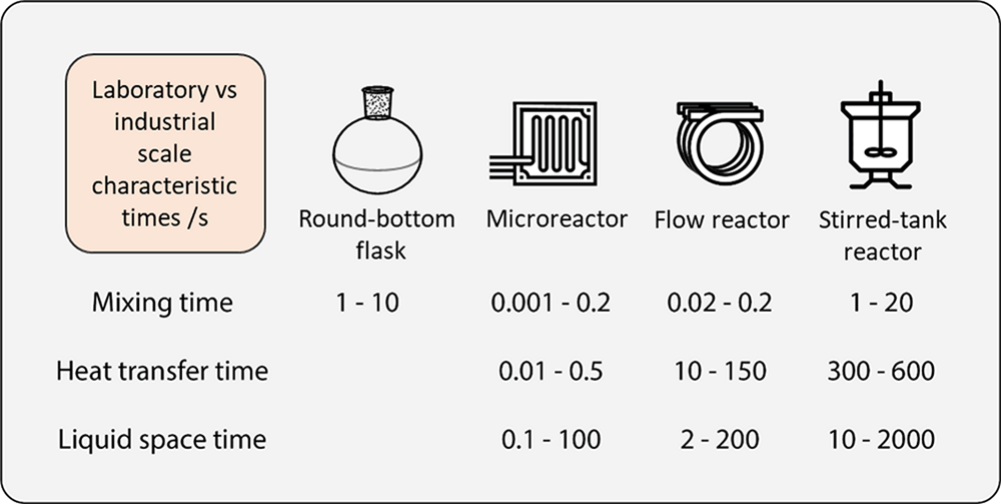
Laboratory vs industrial scale: comparison of characteristic times (in seconds) for mixing, heat transfer and liquid space time observed in reactors used in benchtop optimization and large-scale industrial reactors.^306,307^

In most chemical applications, the design of a large-throughput process involves a multifold increase in the size of the reactor vessels that were used for kinetic or optimization studies in the laboratory environment. The processing environment is likely to drastically change; this is because of significant reductions in surface area/volume ratios that imposes limitations on heat transfer rate, sensitivity to mixing, and different time of addition and removal of products.^306^Figure 20 summarizes the typical time ranges of characteristic mixing, heat transfer, and liquid space time (reactor volume divided per volumetric flow rate) for different reactors used in academia and industry (shake flask, flow reactors, microreactors, stirred-tank reactors). During the transition to the large-scale reactors, mixing and heat transfer become several orders of magnitude slower. While this imposes little or no consequences for slow reactions, which can be scaled-up in a relatively straightforward manner,^303^ for fast reactions the extended time necessary to mix phases in a large-reactor results in limited chemical availability of each reactant leading to suboptimal outputs.

Therefore, finding the optimal reaction conditions for a scaled-up process cannot rely on a simple linear increase of the reactant feed and processing time from the optimal lab benchmark. From a process design perspective, the availability of the intrinsic kinetic model can significantly accelerate process development,^308^ and the ability to generate such models with automated flow methods has been highlighted as a potential paradigm shift in the process systems engineering community.^162^ Notably, such models should be obtained with no mass/heat/mixing limitations. To design kinetic determination experiments unhindered by these effects, Jensen and co-workers^309^ provided a simplified chart-based method for the evaluation of mixing and dispersion in small-scale flow systems, while detailed insights into overcoming heat transfer limitation during optimization in flow reactors were discussed by Mase and co-workers.^310^

Although kinetic models are a powerful tool for accelerating process scale-up, in some cases, the experimental effort necessary to derive these models is extensive, making it infeasible to realize in the fast-paced process development environment. In such cases, Stitt and Simmons^303^ recommend determination from benchtop optimization, at a minimum, the following information: (i) reaction network and the significant byproducts, (ii) if the reaction kinetics follow simple power-laws, (iii) sensitivity of the reaction selectivity to mass transfer effects, and (iv) heat evolution and the potential for reaction runaway. Practical guidelines toward the use of flow chemistry setup for heat measurements were proposed by Meier and co-workers,^311^ while Bourne and co-workers^312^ discussed best practices toward continuous-flow aided kinetic analysis. Other, case-specific strategies for extracting meaningful information for process scale-up from optimization studies are summarized in the reviews of industrial practices in large-scale deployment of novel reactive routes,^313^ such as photoredox catalysis,^314,315^ electrochemistry,^316^ C–H activation,^317^ reductive coupling,^318^ and flow chemistry.^319^

Another aspect to address within optimization studies is the preferred production mode for the scaled-up process, as the rationale for its choice may differ between early benchtop experiments and large-scale production, as broadly described by Trout and co-workers.^320^ In a batch process, feedstocks are supplied all at once to the reactor, and the treatment of the subsequent load of feed materials starts only when the previous batch is fully processed and removed. Batch processing is frequently preferred due to its simplicity and is particularly convenient in pharmaceutical processes that require frequent cleaning of used equipment, or even removal and disposal of single-use reactors between batches (e.g., biomanufacturing lines^321^). However, the design simplicity comes at an extra cost of slow processing, increased energy consumption for start-up and shutdown of each batch, significant environmental impact from cleaning solvents, and more challenging process parameters control. Continuous processing, in contrast, allows for a significant cost and environmental footprint reduction (if solvents are recycled^322,323^) by faster conversion and increased productivity, reduced down-time, and improved quality by facilitating continuous monitoring of critical parameters. From the perspective of the chemist, it is important to be aware of the preferred processing mode and consider process limitations related to each mode within the design of lab-scale experiments.

Within the pharmaceutical and fine chemical industries, the ability to fulfill quality-critical parameters is one of the key goals of process development, but these aspects are typically not investigated before moving toward scale-up studies. Most recent industrial practices, described by Tsai and co-workers,^324^ describe how to systematically include quality considerations across all phases, including reaction optimization.

#### 7.1.1 Classical Approaches Toward Scale-Up

After transferring the data from benchtop optimization to process development, models of scaled-up reactors are obtained to account for engineering phenomena, e.g., characterization of heat and mass transfer environments. If detailed kinetic models are available, the engineer can simulate the operation of a large-scale process and determine a new optimal set of conditions. Gatica and co-workers provided a detailed overview of the industrial practices for the development of design equations to model such processes,^325^ whereas a review by Patterson^326^ addresses the challenges in modeling of mixing- and temperature-sensitive chemical reactions at larger scales. Other reports from the literature also provide insightful examples of computational fluid dynamics (CFD) use for modeling of mixing phenomena.^327,328^

Scale-up engineers seek to quantify the influence of heat and mass transfer, as well as other physical properties, on reaction progression. This also includes the interactions between each physicochemical property. This problem is frequently simplified by dimensional analysis that enables scientists to develop relations among physical quantities (e.g., velocity, viscosity, surface tension) using their dimensions expressed in base units (combination of, e.g., meter, kilogram, second). Detailed instructions on the derivation and use of dimensional analysis equations, followed by practical examples of calculations for stirred-tank reactors, were provided by Zlokarnik^329,330^ and Wild and co-workers.^331^ These equations enable an engineer to disregard the parameters that are not critical for the given reaction, and further develop the nonlinear relationships guiding scale-up. If necessary, experiments to determine the significance of these variables for successful scale-up can be designed. The experiments can be planned for a *partial similarity* or *complete similarity* approach, and advantages and limitations of both approaches are described by Kind and co-workers^332^ and illustrated by the case study of a scale-up of competitive chemical reactions.

Another aspect addressed at the scale-up stage is the choice of a reactor. While stirred-tank or tubular reactors are by far the most deployed reactors in the industrial setting, development of the process intensification field^333^ yields a wide variety of novel reactors offering designs that can drastically enhance heat and mass transfer as well as increase the controllability of reactants and product spatial concentrations distributions. The choice of the reactor also includes insights into construction materials and their interactions with the reaction medium, compliance with the regulatory requirements for the given final product, and the lead manufacturing time. Guidelines provided by Moran and Henkel^334^ compare the reactors typically used in industry; the toolbox proposed by Roberge and co-workers includes both the choice of processing mode and the reactor,^335^ while the contribution of Lindeque^336^ specifically addresses reactor considerations in biocatalytic production of pharmaceuticals compounds; Dautzenberg and Mukherjee^337^ discuss the choice and deployment of multifunctional reactors; West and co-workers^338^ focus on scalable autothermal reactors. Methods for selection between a wider range of novel, process intensification reactors were proposed by Commenge and Falk,^306^ and an overview of different options was presented in an open-source database published by Gorak and co-workers.^339^

Selected reactor architectures and the optimal conditions determined for a scaled-up process are frequently verified on a scale of pilot or mini-plant operation, whereas operability and stability are tested in pilot plants. Industrial practice proves that this intermediate step in process development has the substantial potential to improve process understanding and consequently product quality and operational safety, however, it also increases time-to-market and overall project costs. Design of such plants was described in detail by Whalley.^340^ Examples of scale-up toward a pilot/manufacturing plant capacity are available in the literature, including a hydrazine condensation study by Lane and co-workers and the edaravone synthesis by Sun and co-workers.^341,342^

#### 7.1.2 Alternative Approaches: Numbering Up

The discussed strategies for process scale-up include both extensive experimental insights on laboratory scale, simultaneous modeling of numerous phenomena, and potential pilot plant testing. Despite the availability of the methodological tools described above, it still may not be possible to reproduce the selectivity achieved in benchtop tests in the scale-up setting, particularly if the optimization studies were conducted in microreactors that offer excellent heat and mass transfer characteristics. In such cases, increases in the plant throughput can be achieved instead by the numbering-up approach, which involves the simultaneous use of hundreds to thousands of reactors of the same or similar scale to the ones used in process optimization studies.^158^ This enables one to achieve exactly the same conditions as found in the lab, improves safety by better temperature control and drastically reduces scale-up time. Possibilities of the accelerated process development by numbering-up of microreactors are covered in detail by Kockmann and co-workers^343^ and Roberge and co-workers.^344^ However, numbering-up is associated with high investment cost: numerous reactors are required instead of a single stirred-tank reactor, along with multiple process control devices.

Detailed insights into the economics of numbering-up were described by Weber and Snowden-Swan,^345^ and the potential to intensify the process can be evaluated by the process intensification score proposed by van der Meer and co-workers.^346,347^ Capital costs considerations mean that this approach can be economically justified only for high-end products, and for cases where a drastic improvement of output (e.g., reaction yield) has been demonstrated. Interestingly, an analysis conducted by the Process Development Team from Lonza^348^ reveals that 50% of reactions in the fine chemical/pharmaceutical industry could benefit from the deployment of microreactor technology. Lowe and co-workers^349^ proposed a benchmarking method allowing for a similar evaluation across different industries. Notably, as the economic factors are limiting the deployment of microreactors, their market availability is also lower, and the extended lead time could be a key limitation for decision makers that require fast and large product delivery. By more frequent adoption of novel process optimization methods that involve the use of microreactors, we anticipate the unveiling of more case studies where excellent optima are achievable solely by this method, and hence, we can expect accelerated adoption of more efficient process intensification technologies.

### 7.2 Safety Considerations

For both scaling- and numbering-up approaches, in batch or continuous mode, there are several safety concerns to be addressed. Large volumes of flammable solvent are almost always required, meaning that precautions must be taken to minimize the risk of fire from static electricity build up or exposed flame. However, the greatest concern with organic processes is thermal runaway.^350^ It is important to consider these physical limitations when running optimization campaigns, as algorithms or experimental designs may suggest experiments that are out of safe operating bounds: this includes reactions that are suggested at unsafe temperatures or concentrations.

Thermal runaway occurs when the rate of an exothermic reaction is accelerated by increases in temperature. In the worst case, this rate acceleration can lead to secondary decomposition reactions that are more energetic and hazardous than the primary synthesis. While reactions commonly used in fine chemicals such as the Suzuki–Miyaura cross coupling are often not thought to be dangerously exothermic at the bench, they can have significant exotherms that could lead to thermal runaway at scale.^351^ The exotherms of reactions need to be understood via calorimetry studies in the lab, so that control strategies can be implemented during scale-up.

Reaction calorimetry enables the measurement of the heat produced by a reaction over time. Often, two parameters are determined: the adiabatic temperature rise (Δ*T*_adia_) and the maximum temperature of synthesis reaction (MTSR). Δ*T*_adia_ is the temperature rise in a specific volume for a specific reaction when all the heat of reaction is delivered to an adiabatic system (i.e., no heat is transferred between the system and its surroundings). MTSR is defined as the maximum temperature an adiabatic reactor would reach if cooling failed. In other words, MSTR is the sum of the reaction temperature *T*_p_ and Δ*T*_adia_. Together, Δ*T*_adia_ and MTSR can give insights of the worst-case scenario for a particular reaction and reactor. For example, Wang and co-workers conducted calorimetry studies of copper-mediated fluorinations of bromopyridines and found that the MTSR was above the decomposition of the chosen reaction solvent, DMSO.^352^ Because decomposing DMSO is an explosion hazard,^353,354^ control strategies such as reduced speed dosing or switching to an alternative solvent needed to be implemented.

Readers interested in learning more about the safety considerations for reaction development are referred to the excellent reviews by Yang on safety aspects of DMSO^354^ and Pd-catalyzed reactions,^350^ as well as the textbook by Stoessel on “*Thermal Safety of Chemical Processes*”.^355^

## 8 Conclusion

In this review, we have outlined several modern techniques that are utilized for chemical reaction optimization to serve as an accessible reference for interested bench scientists. We have also given discussions on their relationship to further stages of process development, namely scale-up. There are many distinct methodologies that can be used to obtain optimal reactions conditions for desired outcomes (reaction yield, selectivity, *E*-factor, etc.), and there are trade-offs to consider for research organizations when deciding which to implement. These decisions must balance the costs associated with each technique (equipment costs, training costs, time investments) with the deliverables that they hope to achieve and their associated accuracy and reliability. As automated equipment is becoming more ubiquitous and user-friendly, this is one possible solution to unify several fields (chemistry, process engineering, computer science) to conduct reaction optimization techniques in novel, systematic ways to maximize process outputs. This could be in conducting automated DoE or kinetic studies, screening every combination of reaction variables in HTE or utilizing self-optimization, or more!

Researchers may willingly run intuition-driven experimentation, even when knowing of more effective techniques, as the novelty of their research may focus on other aspects of chemistry (such as reaction discovery, substrate scopes, etc.) rather than complete optimization of their processes. However, as modern laboratories are becoming more diversified in skillsets and more interdisciplinary research is conducted, familiarity with these techniques must be embraced and undergraduate/postgraduate courses will undoubtedly reflect this more in coming years. It is easy to envision an evolving chemistry course with practical modules in DoE, HTE, and more, as the skillsets of chemists diversify beyond traditional synthesis to meet the needs of the modern laboratory. It is the hope that this timely review will prove the accessibility of these optimization techniques and help to encourage inspired chemists to incorporate them into their workflows.
